# Racial and ethnic differences in prenatal exposure to environmental phenols and parabens in the ECHO Cohort

**DOI:** 10.1038/s41370-025-00750-w

**Published:** 2025-02-15

**Authors:** Michael S. Bloom, Sudhi Upadhyaya, Adaeze W. Nzegwu, Jordan R. Kuiper, Jessie P. Buckley, Judy Aschner, Dana Barr, Emily S. Barrett, Deborah H. Bennett, Dana Dabelea, Anne L. Dunlop, Alma Fuller, Margaret Karagas, Donghai Liang, John Meeker, Rachel Miller, Thomas G. O’Connor, Megan E. Romano, Sheela Sathyanarayana, Anne P. Starling, Annemarie Stroustrup, Deborah J. Watkins

**Affiliations:** 1 Department of Global and Community Health, College of Public Health, George Mason University, Fairfax, VA USA; 2 Department of Epidemiology, Johns Hopkins Bloomberg School of Public Health, Baltimore, MD USA; 3 Department of Environmental and Occupational Health, Milken Institute School of Public Health, The George Washington University, Washington, DC USA; 4 Department of Epidemiology, Gillings School of Global Public Health, University of North Carolina at Chapel Hill, Chapel Hill, NC USA; 5 Hackensack Meridian Health Center for Discovery and Innovation, Hackensack, NJ USA; 6 Gangarosa Department of Environmental Health, Rollins School of Public Health, Emory University, Atlanta, GA USA; 7 Department of Biostatistics and Epidemiology, Rutgers School of Public Health, and Environmental and Occupational Health Sciences Institute, Rutgers University, Piscataway, NJ USA; 8 Department of Public Health Sciences, School of Medicine, University of California, Davis, Davis, CA USA; 9 Department of Epidemiology, University of Colorado, Colorado School of Public Health, Aurora, CO USA; 10 Lifecourse Epidemiology of Adiposity and Diabetes (LEAD) Center, University of Colorado Anschutz Medical Campus, Aurora, CO USA; 11 Department of Gynecology and Obstetrics, Emory University School of Medicine, Atlanta, GA USA; 12 School of Nursing, College of Public Health, George Mason University, Fairfax, VA USA; 13 Department of Epidemiology, Geisel School of Medicine at Dartmouth, Lebanon, NH USA; 14 Department of Environmental Health Sciences, University of Michigan School of Public Health, Ann Arbor, MI USA; 15 Division of Clinical Immunology, Icahn School of Medicine at Mount Sinai, New York, NY USA; 16 Departments of Psychiatry, Neuroscience, Obstetrics and Gynecology, University of Rochester, Rochester, NY USA; 17 Department of Pediatrics, University of Washington, Seattle, WA USA; 18 Northwell Health, Cohen Children’s Medical Center and the Departments of Pediatrics and Occupational Medicine, Epidemiology & Prevention, Zucker School of Medicine at Hofstra/Northwell, New Hyde Park, NY USA; 19 Division of Neonatology, Department of Pediatrics, Duke Clinical Research Institute, Duke University School of Medicine, Durham, NC USA; 20 Division of Cardiology, Department of Medicine, Duke Clinical Research Institute, Duke University School of Medicine, Durham, NC USA; 21 Department of Nutrition, Gillings School of Global Public Health, University of North Carolina at Chapel Hill, Chapel Hill, NC USA; 22 Department of Epidemiology, Johns Hopkins University, Bloomberg School of Public Health, Baltimore, MD USA; 23 Research Triangle Institute, Research Triangle Park, NC USA; 24 Department of Medical Social Sciences, Feinberg School of Medicine, Northwestern University, Chicago, IL USA; 25 Department of Pediatrics, Feinberg School of Medicine, Northwestern University and Ann & Robert H. Lurie Children’s Hospital of Chicago, Chicago, IL USA; 26 Division of Infectious Diseases, Department of Medicine, Vanderbilt University Medical Center, Nashville, TN USA; 27 Division of Cardiovascular Medicine, Department of Medicine, Vanderbilt University Medical Center, Nashville, TN USA; 28 Department of Chemistry, Vanderbilt University, Nashville, TN USA; 29 College of Engineering, Northeastern University, Boston, MA USA; 30 College of Public Health, Department of Epidemiology & Biostatistics, University of Georgia, Athens, GA USA; 31 Environmental Health Sciences, School of Public Health, University of Michigan, Ann Arbor, MI USA; 32 Departments of Pediatrics and Population Health, NYU Grossman School of Medicine, New York, NY USA; 33 Department of Emergency Medicine, Massachusetts General Hospital, Harvard Medical School, Boston, MA USA; 34 Environmental and Radiological Health Sciences, Colorado School of Public Health, Colorado State University, Fort Collins, CO USA; 35 Epidemiology, University of North Carolina at Chapel Hill, Chapel Hill, NC USA; 36 Center for Health Systems Research, Denver Health and Hospital Authority, Denver, CO USA; 37 Department of Pediatrics, Rhode Island Hospital, The Alpert Medical School of Brown University, Providence, RI USA; 38 Division of Gender Equality, Maternal, Newborn & Child Health Discovery & Tools Team, Bill & Melinda Gates Foundation, Seattle, WA USA; 39 Department of Statistics, University of California, Davis, Davis, CA USA; 40 Division of Child and Adolescent Psychiatry, Columbia University—NYSPI, New York, NY USA; 41 Department of Obstetrics & Gynecology, Columbia University—NYSPI, New York, NY USA; 42 Behavioral Sciences Research Institute, University of Puerto Rico, School of Medicine, Rio Piedras, Puerto Rico; 43 Child & Family Mental Health & Community Psychiatry Division, Duke University School of Medicine, Duke Psychiatry & Behavioral Sciences, Durham, NC USA; 44 Department of Psychology, Emory University, Atlanta, GA USA; 45 Avera Institute, Rapid City, SD USA; 46 Department of Pediatrics, University of South Dakota School of Medicine, Rapid City, SD USA; 47 Department of Pediatrics, Avera Research Institute, University of South Dakota School of Medicine, Sioux Falls, SD USA; 48 Division of Research, Kaiser Permanente Northern California, Oakland, CA USA; 49 Centre for Health Informatics, University of Manchester, Manchester, United Kingdom; 50 Department of Pediatrics, Monroe Carell Jr Children’s Hospital at Vanderbilt, Vanderbilt University Medical Center, Nashville, TN USA; 51 National Farm Medicine Center, Marshfield Clinic Research Institute, Marshfield, WI USA; 52 Department of Pediatrics, University of Wisconsin School of Medicine and Public Health, Madison, WI USA; 53 The Channing Division of Network Medicine; Department of Medicine, Brigham and Women’s Hospital, Harvard Medical School, Boston, MA USA; 54 Division of Pediatric Allergy, Immunology, and Pulmonary Medicine, Department of Medicine, Department of Pediatrics, Vanderbilt University Medical Center, Nashville, TN USA; 55 Department of Public Health Sciences, Henry Ford Health, Detroit, MI USA; 56 Department of Pediatrics, Columbia University Medical Center, New York, NY USA; 57 Division of Asthma Research, Cincinnati Children’s Hospital Medical Center, Cincinnati, OH USA; 58 Department of Medicine, University of California, San Francisco, CA USA; 59 Department of Medicine; Division of Clinical Immunology, Icahn School of Medicine at Mount Sinai, New York, NY USA; 60 Department of Pediatrics, Boston University School of Medicine, Boston, MA USA; 61 Department of Human Genetics, University of Chicago, Chicago, IL USA; 62 Department of Pediatrics, Washington University School of Medicine, St Louis, MO USA; 63 Department of Pediatrics and College of Medicine; Division of Biostatistics and Epidemiology, University of Cincinnati, Cincinnati, OH USA; 64 Department of Pediatrics, Johns Hopkins University School of Medicine, Baltimore, MD USA; 65 Division of Allergy and Clinical Immunology, Henry Ford Health, Detroit, MI USA; 66 Department of Population and Public Health Sciences, University of Southern California, Los Angeles, CA USA; 67 MIND Institute and Department of Public Health Sciences, University of California, Davis, Davis, CA USA; 68 Department of Public Health Sciences, University of California, Davis, Davis, CA USA; 69 Department of Psychiatry and Behavioral Science and the MIND Institute, University of California, Davis, Davis, CA USA; 70 Medical Microbiology and Immunology; MIND Institute, University of California, Davis, Davis, CA USA; 71 Psychiatry and Psychology, University of Pittsburgh, Pittsburgh, PA USA; 72 Psychiatry and Behavioral Neuroscience, University of Chicago, Chicago, IL USA; 73 Department of Pediatrics, School of Medicine; Department of Environmental and Occupational Health Sciences; School of Public Health, University of Washington, Seattle, WA USA; 74 Department of Psychiatry and Behavioral Sciences and Department of Pediatrics, School of Medicine, University of California, San Francisco, San Francisco, CA USA; 75 Department of Psychiatry and Behavioral Sciences, School of Medicine, University of California, San Francisco, San Francisco, CA USA; 76 Department of Pediatrics, School of Medicine; Department of Environmental and Occupational Health Sciences, School of Public Health, University of Washington and Seattle Children’s Research Institute, Seattle, WA USA; 77 Department of Preventive Medicine, University of Tennessee Health Science Center, Memphis, TN USA; 78 Department of Pediatrics, Department of Environmental Medicine & Public Health, Icahn School of Medicine at Mount Sinai, New York, NY USA; 79 Department of Environmental and Occupational Health Sciences; School of Public HealthUniversity of Washington, Seattle, WA USA; 80 Department of Counseling Psychology and Human Services & Prevention Science InstituteUniversity of Oregon, Eugene, OR USA; 81 Department of Psychological and Behavioral Sciences, George Washington University, Washington, DC, USA; 82 Department of Psychology, Penn State University, University Park, PA USA; 83 Channing Division of Network Medicine, Department of Medicine, Brigham and Women’s Hospital and Harvard Medical School, Boston, MA USA; 84 Pediatric Pulmonary Division, Department of Pediatrics, Golisano Children’s Hospital, University of Rochester, Rochester, NY USA; 85 Division of Neonatology, Department of Pediatrics, Oregon Health & Science University, Portland, OR USA; 86 Division of Neuroscience, Oregon National Primate Research Center, Beaverton, OR USA; 87 Division of Pediatric Pulmonology, Department of Pediatrics, Indiana School of Medicine, Indianapolis, IN USA; 88 College of Health and Human Development, Penn State, State College, PA USA; 89 AJ Drexel Autism Institute, Drexel University, Philadelphia, PA USA; 90 Mental Health, Johns Hopkins University, Baltimore, MD USA; 91 Department of Psychiatry and Behavioral Sciences, Center for Autism and Related Disorders, Kennedy Krieger Institute, Johns Hopkins University, Baltimore, MD USA; 92 MIND Institute, Department of Psychiatry, University of California Davis, Sacramento, CA USA; 93 Department of Psychiatry, University of North Carolina, Chapel Hill, NC USA; 94 Center for Autism Research, Children’s Hospital of Philadelphia, Philadelphia, PA USA; 95 Department of Radiology, University of Washington, Seattle, WA USA; 96 Department of Psychiatry, Washington University, St Louis, MO USA; 97 Department of Psychology, University of Miami, Miami, FL USA; 98 Department of Psychology, University of Washington, Seattle, WA USA; 99 Kaiser Permanente Division of Research, Kaiser Permanente, Oakland, CA USA; 100 Departments of Obstetrics and Gynecology, University of Rochester, Rochester, NY USA; 101 Division of Chronic Disease Research Across the Lifecourse, Department of Population Medicine, Harvard Pilgrim Health Care Institute and Harvard Medical School, Boston, MA USA; 102 Department of Obstetrics and Gynecology, Beth Israel Deaconess Medical Center, Boston, MA USA; 103 Department of Environmental Health, Harvard Chan School of Public Health, Boston, MA USA; 104 Division of Neonatology, Department of Pediatrics, University of North Carolina School of Medicine, Chapel Hill, NC USA; 105 Department of Environmental Sciences and Engineering, University of North Carolina Gillings School of Global Public Health, Chapel Hill, NC USA; 106 EK Shriver Center and Psychiatry, UMASS Chan Medical School, Worcster, MA USA; 107 Department of Pediatrics, Tufts University School of Medicine, Boston, MA USA; 108 Department of Neurology, Harvard Medical School, Boston, MA USA; 109 Division of Neonatology, Department of Pediatrics, Yale School of Medicine, New Haven, CT USA; 110 Department of Pediatrics, University of Massachusetts Chan Medical School-Baystate, Springfield, MA USA; 111 Department of Anatomy & Neurobiology, Boston University Chobanian & Avedisian School of Medicine, Boston, MA USA; 112 Pediatrics, Wake Forest School of Medicine, Winston-Salem, NC USA; 113 Section of Neonatology, Department of Pediatrics, Wake Forest School of Medicine, Wake Forest University School of Medicine/Atrium Health Wake Forest, Winston-Salem, NC USA; 114 Section of Neonatology, Department of Pediatrics, ECU Health, Greenville, NC USA; 115 Department of Health Sciences, School of Medicine, University of North Carolina at Chapel Hill, Chapel Hill, NC USA; 116 Pediatrics, School of Medicine, University of North Carolina at Chapel Hill, Chapel Hill, NC USA; 117 Department of Anthropology, Department of Nutrition, University of North Carolina at Chapel Hill; Gillings School of Global Public Health, University of North Carolina at Chapel Hill, Chapel Hill, NC USA; 118 Epidemiology and Maternal and Child Health, University of North Carolina at Chapel Hill; Gillings School of Global Public Health, University of North Carolina at Chapel Hill, Chapel Hill, NC USA; 119 Environmental Sciences and Engineering, Gillings School of Global Public Health, University of North Carolina at Chapel Hill, Chapel Hill, NC USA; 120 Kennedy Research Center on Intellectual and Neurodevelopmental Disabilities, University of Chicago Medicine: Comer Children’s Hospital, Chicago, IL USA; 121 Department of Epidemiology and Biostatistics, Michigan State University, East Lansing, MI USA; 122 Pediatrics, Beaumont Hospital, Royal Oak, MI USA; 123 Pediatrics, Corewell Health, Helen DeVos Children’s Hospital, Grand Rapids, MI USA; 124 Epidemiology and Prevention, Wake Forest University School of Medicine, Winston-Salem, NC USA; 125 Pediatrics, Mass General Hospital for Children, Boston, MA USA; 126 Dean’s Office Graduate School, School of Nursing and Health Studies, University of Miami, Coral Gables, FL USA; 127 Departments of Epidemiology & Biostatistics, and Pediatrics & Human Development, Michigan State University, College of Human Medicine, East Lansing, MI USA; 128 Department of Pediatrics, Henry Ford Health, Detroit, MI USA; 129 Department of Biostatistics, University of MI, Ann Arbor, MI USA; 130 Department of Obstetrics and Gynecology, Institute of Environmental Health Sciences (IEHS), C.S. Mott Center for Human Health and Development, Wayne State University, Detroit, MI USA; 131 Lifecourse Epidemiology and Genomics Division, Michigan Department of Health and Human Services (MDHHS), Lansing, MI USA; 132 Department of Environmental Health Sciences, Columbia University Mailman School of Public Health, New York, NY USA; 133 Department of Psychiatry, Columbia University Irving Medical Center, New York, NY USA; 134 Beckman Institute for Advanced Science and Technology; Department of Comparative Biosciences, University of Illinois Urbana-Champaign, Urbana, IL USA; 135 Beckman Institute for Advanced Science and Technology; Department of Kinesiology and Community Health, University of Illinois Urbana-Champaign, Urbana, IL USA; 136 Beckman Institute for Advanced Science and Technology; Department of Social Work, University of Illinois Urbana-Champaign, Urbana, IL USA; 137 Department of Food Science and Human Nutrition, Michigan State University, East Lansing, MI USA; 138 Program on Reproductive Health and the Environment, University of California, San Francisco, San Francisco, CA USA; 139 Department of Environmental Science, Policy and Management and School of Public Health, University of California, Berkeley, Berkeley, CA USA; 140 Department of Family and Preventive Medicine, Spencer Fox Eccles School of Medicine, University of Utah, Salt Lake City, UT USA; 141 Department of Pediatrics, Spencer Fox Eccles School of Medicine, University of Utah, Salt Lake City, UT USA; 142 Department of Environmental Medicine & Public Health, Icahn School of Medicine at Mount Sinai, New York, NY USA; 143 Department of Psychiatry and Behavioral Medicine, University of Washington, Seattle Children’s Research Institute, Seattle, WA USA; 144 Department of Population Health Sciences, University of Wisconsin, Madison, WI USA; 145 Department of Statistics, University of Pittsburgh, Pittsburgh, PA USA; 146 Department of Medicine, University of Washington, Seattle, WA USA; 147 Department of Pediatrics, Albert Einstein College of Medicine, Bronx, NY USA; 148 Center for Discovery and Innovation, Hackensack Meridian Healthcare, Nutley, NJ USA; 149 Department of Pediatrics, Northwell Health, Cohen Children’s Medical Center, and the Zucker School of Medicine at Hofstra/Northwell, New Hyde Park, NY USA; 150 Department of Pediatrics, Cincinnati Children’s, Cincinnati, OH USA; 151 Department of Pediatrics, Vanderbilt University Medical Center, Nashville, TN USA; 152 Department of Pediatrics, University of Rochester Medical Center, Rochester, NY USA; 153 Department of Pediatrics, University of Florida College of Medicine, Jacksonville, FL USA; 154 Department of Pediatrics, University of Buffalo Jacobs School of Medicine and Biomedical Sciences, Buffalo, NY USA; 155 Department of Pediatrics, Children’s Minnesota, Minneapolis, MN USA; 156 Department of Obstetrics and Gynecology, Northwell Health and the Zucker School of Medicine at Hofstra / Northwell, New Hyde Park, NY USA; 157 Department of Science Education, Northwell Health and the Zucker School of Medicine at Hofstra/Northwell, New Hyde Park, NY USA; 158 Institute of Health System Science, Northwell Health, Feinstein Institutes for Medical Research, Manhasset, NY USA; 159 Department of Pediatrics, Children’s Healthcare of Atlanta Emory University, Atlanta, GA USA; 160 Department of Biostatistics, Vanderbilt University Medical Center, Nashville, TN USA; 161 Department of Obstetrics and Gynecology, Vanderbilt University Medical Center, Nashville, TN USA; 162 Department of Women’s Health, Henry Ford Health, Detroit, MI USA; 163 Department of Biostatistics and Epidemiology, Environmental and Occupational Health Sciences Institute, Rutgers University, Piscataway, NJ USA; 164 Center for Advanced Biotechnology & Medicine, Rutgers University, Piscataway, NJ USA; 165 Departments of Biochemistry and Microbiology & Anthropology, Rutgers University, New Brunswick, NJ USA; 166 Department of Pediatrics, Robert Wood Johnson Medical School, Rutgers University, New Brunswick, NJ USA; 167 Departments of Pediatrics, Family Medicine, and Community Health, Robert Wood Johnson Medical School, Rutgers University, New Brunswick, NJ USA; 168 Department of Obstetrics, Gynecology, and Reproductive Sciences, Robert Wood Johnson Medical School, Rutgers University, New Brunswick, NJ USA; 169 Department of Obstetrics and Gynecology, Saint Peter’s University Hospital, New Brunswick, NJ USA; 170 Department of Public Health Sciences, Medical University of South Carolina, Charleston, SC USA; 171 Department of Obstetrics and Gynecology, Medical University of South Carolina, Charleston, SC USA; 172 Department of Global and Community Health, George Mason University, Fairfax, VA USA; 173 Department of Pediatrics, Medical University of South Carolina, Charleston, SC USA; 174 Department of Biostatistics, Epidemiology and Informatics; Department of Obstetrics and Gynecology, University of Pennsylvania Perelman School of Medicine, Philadelphia, PA USA; 175 Division of Neonatology, Department of Pediatrics, Children’s Hospital of Philadelphia, University of Pennsylvania Perelman School of Medicine, Philadelphia, PA USA; 176 Division of Maternal-Fetal Medicine, Department of Obstetrics & Gynecology, Feinberg School of Medicine, Northwestern University, Chicago, IL USA; 177 Division of Neonatology, Department of Pediatrics, Ann & Robert H. Lurie Children’s Hospital, Feinberg School of Medicine, Northwestern University, Chicago, IL USA; 178 Division of Maternal-Fetal Medicine, Department of Obstetrics & Gynecology, Hackensack University Medical Center, Hackensack Meridian School of Medicine, Nutley, NJ USA; 179 Division of Neonatology, Department of Pediatrics, Hackensack University Medical Center, Hackensack Meridian School of Medicine, Nutley, NJ USA; 180 Division of Developmental and Behavioral Pediatrics, Department of Pediatrics, Hackensack University Medical Center, Hackensack Meridian School of Medicine, Nutley, NJ USA; 181 Department of Pathology, Feinberg School of Medicine, Northwestern University, Chicago, IL USA; 182 Division of Infectious Diseases, Department of Pediatrics, Ann & Robert H. Lurie Children’s Hospital, Feinberg School of Medicine, Northwestern University, Chicago, IL USA; 183 Division of Neonatology, Department of Pediatrics, Emory University School of Medicine, Atlanta, GA USA; 184 Cerebral Palsy Foundation, New York, NY USA; 185 Division of Epidemiology & Community Health, School of Public Health, University of Minnesota, Minneapolis, MN USA; 186 Division of Research & Evaluation, HealthPartners Institute, Minneapolis, MN USA; 187 Care Delivery Research, Allina Health, Minneapolis, MN USA; 188 Department of Obstetrics and Gynecology, University of Miami Miller School of Medicine, Miami, FL USA; 189 Department of Obstetrics, Gynecology and Reproductive Sciences, University of Miami Miller School of Medicine, Miami, FL USA; 190 Mailman Center for Child Development, University of Miami Miller School of Medicine, Miami, FL USA; 191 Department of Obstetrics, Gynecology and Reproductive Sciences and Department of Public Health Sciences, University of Miami School of Medicine, Miami, FL USA; 192 Department of Pediatrics, University of Miami Miller School of Medicine, Miami, FL USA; 193 School of Nursing and Health Studies, University of Miami, Miami, FL USA; 194 Psychiatry and Behavioral Sciences; MIND Institute, University of California Davis, Sacramento, CA USA; 195 Center for Perinatal Research, Abigail Wexner Research Institute and Division of Neonatology, Nationwide Children’s Hospital and Department of Pediatrics, College of Medicine and Division of Epidemiology, College of Public Health, The Ohio State University, Nationwide Children’s Hospital and The Ohio State University, Columbus, OH USA; 196 Center for Biobehavioral Health, Abigail Wexner Research Institute, Nationwide Children’s Hospital and Department of Pediatrics, College of Medicine and Division of Epidemiology, College of Public Health, The Ohio State University, Nationwide Children’s Hospital and The Ohio State University, Columbus, OH USA; 197 Division of Maternal-Fetal Medicine, Department of Obstetrics and Gynecology, College of Medicine and Division of Epidemiology, College of Public Health, The Ohio State University, The Ohio State University, Columbus, OL USA; 198 Center for Precision Environmental Health and Department of Medicine, Baylor College of Medicine, Houston, TX USA; 199 Department of Family and Community Medicine, University of Texas Health Science Center at Houston (UTHealth Houston) McGovern Medical School, Houston, TX USA; 200 Department of Obstetrics, Gynecology and Reproductive Sciences, University of Texas Health Science Center at Houston (UTHealth Houston) McGovern Medical School, Houston, TX USA; 201 Department of Pediatrics, University of Texas Health Science Center at Houston (UTHealth Houston) McGovern Medical School, Houston, TX USA; 202 Department of Epidemiology, Geisel School of Medicine at Dartmouth, Hanover, NH USA; 203 Departments of Psychiatry, Pediatrics & Epidemiology, Geisel School of Medicine at Dartmouth, Dartmouth Hitchcock Medical Center, Hanover, NH USA; 204 Community Environmental Health Program, Department of Pharmaceutical Sciences, College of Pharmacy, University of New Mexico Health Sciences Center, Albuquerque, NM USA; 205 Center for Development and Disability, University of New Mexico, Albuquerque, NM USA; 206 Community Environmental Health Program, Department of Pharmaceutical Sciences, College of Pharmacy, University of New Mexico Health Sciences Center, Albuquerque, NM USA; 207 University of Chicago, Chicago, IL USA; 208 Department of Psychiatry and Behavioral Sciences, University of California, San Francisco, San Francisco, CA USA; 209 Department of Internal Medicine, Comprehensive Cancer Center, University of New Mexico Health Sciences Center, Albuquerque, NM USA; 210 Department of Pediatrics, Department of Psychiatry and Human Behavior, Warren Alpert Medical School of Brown University, Providence, RI USA; 211 Department of Environmental Health, Rollins School of Public Health, Emory University, Atlanta, GA USA; 212 Department of Psychiatry and Human Behavior, Warren Alpert Medical School of Brown University, Providence, RI USA; 213 Department of Pediatrics, Warren Alpert Medical School of Brown University, Providence, RI USA; 214 Department of Pediatrics, Thompson Center for Autism & Neurodevelopment, University of Missouri, Columbia, MO USA; 215 Department of Pediatrics, Children’s Mercy-Kansas City, Kansas City, MO USA; 216 Department of Pediatrics, Wake Forest School of Medicine, Winston, Salem, NC USA; 217 Department of Pediatrics, University of Hawaii John A Burns School of Medicine, Honolulu, HI USA; 218 Department of Pediatrics, UCLA Clinical and Translational Science Institute at The Lundquist Institute, Harbor-UCLA Medical Center, Los Angeles, CA USA

**Keywords:** Environmental phenols, Ethnicity, Health inequities, Parabens, Pregnancy

## Abstract

**Background:**

Research suggests racial/ethnic disparities in prenatal exposure to endocrine disrupting environmental phenols (EPs) in limited populations. However, no studies have investigated racial/ethnic disparities in prenatal EP exposure across the U.S.

**Objectives:**

To estimate demographic differences in prenatal urinary EPs among participants in the Environmental influences on Child Health Outcomes (ECHO) Cohort.

**Methods:**

An analysis of 4006 pregnant ECHO participants was performed, with 7854 specimens collected from 1999–2020. Racial/ethnic identity was self-reported. Urinary levels of 2,4-dichlorophenol (2,4-DCP), 2,5-dichlorophenol (2,5-DCP), benzophenone-3 (BP-3), bisphenols A (BPA), F (BPF), and S (BPS), and methyl- (MePb), ethyl- (EtPb), propyl- (PrPb), and butyl- (BuPb) parabens were measured at one or more time points during pregnancy. Effect estimates were adjusted for age, pre-pregnancy body mass index, educational level, gestational age and season at urine collection, and ECHO cohort.

**Results:**

Participants were classified as Hispanic of any race (*n* = 1658), non-Hispanic White (*n* = 1478), non-Hispanic Black (*n* = 490), and non-Hispanic Other (*n* = 362), which included individuals of multiple races. Urinary 2,4-DCP and 2,5-DCP concentrations were 2- to 4-fold higher among Hispanic, non-Hispanic Black, and non-Hispanic Other participants relative to non-Hispanic White participants. MePb was ~2-fold higher among non-Hispanic Black (95% confidence interval (CI): 1.7–3.1) and non-Hispanic Other (95% CI: 1.5–2.8) participants. PrPb was similarly higher among non-Hispanic Black (95% CI: 1.7–3.7) and non-Hispanic Other (95% CI: 1.3–3.1) participants. EtPb was higher among non-Hispanic Black participants (3.1-fold; 95% CI 1.7–5.8). BP-3 was lower in Hispanic (0.7-fold; 95% CI: 0.5–0.9), non-Hispanic Black (0.4-fold; 95% CI: 0.3–0.5), and non-Hispanic Other (0.5-fold; 95% CI: 0.4–0.7) participants. Urinary BuPb, BPA, BPF, and BPS were similar across groups.

**Impact statement:**

This multisite, observational cohort study investigated whether there are racial and ethnic differences in prenatal exposure to endocrine disrupting environmental phenols and parabens. Among 4006 participants from multiple U.S. cohorts who provided urine specimens during pregnancy, those who self-reported a racial and ethnic identity other than non-Hispanic White had higher urinary concentrations of 2,4-dichlorophenol, 2,5-dichlorophenol, methyl paraben, ethyl paraben, and propyl paraben and lower urinary concentrations of benzophenone-3 than those reporting as non-Hispanic White. These data show differences in prenatal concentrations of endocrine disrupting environmental phenols and parabens by racial and ethnic identity.

## Introduction

Gestational exposure to environmental endocrine disrupting chemicals (EDCs) is widespread [1, 2]. Environmental phenols (EPs), including parabens, are types of EDCs with reported estrogenic, anti-androgenic, and thyroid-hormone effects [3]. These chemicals are employed in the manufacture of polycarbonate plastics, food packaging, heat transfer papers like receipts, and medication, among other commercial products, and as ultraviolet filters and preservatives in sunscreens, personal care products, and processed foods as summarized in Supplementary Table 1 [4–8]. Exposure occurs through consumer items, food packaging, personal care products, and household dust [9, 10], and many EPs readily cross the placenta to expose the developing fetus [11]. Despite short in vivo half-lives, EPs are detected frequently in human biospecimens, underscoring their pervasive nature. Prenatal exposure to EPs has been associated with reproductive morbidities, infertility, adverse birth outcomes, altered fetal and child development, and long-term health risks among offspring, possibly partially accounting for poorer reproductive health outcomes among minoritized populations [12–14].

Results of U.S. biomonitoring studies, using data from the National Health and Nutrition Examination Survey, indicate that EP exposure tends to be disproportionately experienced by non-White and low-income groups in the general population [15–19]. Previous studies of urinary EPs among pregnant people in the U.S. have also reported racial, ethnic, and socioeconomic disparities in exposure to EPs [20–24]. Residents of socioeconomically disadvantaged and minoritized communities may experience greater risks of exposure to EPs than advantaged and non-Hispanic White communities, due to greater proximity to industry and waste management facilities, and a limited selection of consumer products and fresh foods [25]. However, these previous studies were limited in size and scope, mostly offering insight into the nature and extent of the exposure disparity on a local basis and/or did not consistently report racial/ethnic differences with adjustment for social determinants. No studies have comprehensively characterized the differences in concentrations of EPs among pregnant people with various self-reported racial and ethnic identities and across different regions of the U.S. [26].

We leveraged extant urinary gestational EP data from 11 cohorts across the U.S. and Puerto Rico within the Environmental Influences on Child Health Outcomes (ECHO) Cohort to help address this important public health data gap. Synthesizing results across multiple studies from different U.S. regions can help inform policy makers on target priorities to eliminate disparities in exposure to EDCs among pregnant populations at a large scale. We selected the EPs for study based on a high reported prevalence of exposure in U.S. study populations, evidence of endocrine disruption, and availability in the ECHO cohorts. We hypothesized that non-White pregnant people would have higher urinary concentrations of most EPs than their White counterparts, conditional on social determinants.

## Methods

### Study participants

The ECHO Cohort consists of mother–offspring pairs in 69 different birth cohorts from across the U.S. [27]. All participants completed written informed consent for participation in their cohorts and consented to data sharing with the ECHO program. We excluded cohorts with <30 eligible participants and participants were required to have at least one urine specimen collected during pregnancy, with laboratory determination of at least one EP, leaving 4139 participants from 11 ECHO cohorts (96.8% were singleton pregnancies, 3% were missing, and 0.2% were multiple gestations). We retained only singleton pregnancies. Thus, a total of 7854 urine specimens from 4006 participants from 11 ECHO cohorts were included in the final analytic sample (Supplementary Figs. 1 and 2; Supplementary Table 2). The study protocol was approved by the single ECHO institutional review board, WIRB Copernicus Group Institutional Review Board.

### Sociodemographic characteristics

Participants self-reported their racial/ethnic identities, which we subsequently categorized as Hispanic of any race, non-Hispanic Black, non-Hispanic White, and non-Hispanic Other—a category that included non-Hispanic Asian, Hawaiian, American Indian, Alaskan Native, multiple races, and other racial identities (the small number of participants in each group precluded statistical analysis of the individual identities). Race is a social construct, used in this analysis as a proxy for individual and systematic lived experiences of racism and discrimination resulting from complex prior and ongoing historical processes based (primarily) on racial grouping [28, 29]. Participants also self-reported their highest completed level of education, used as a proxy for socioeconomic position [30]. Educational level was categorized as ≥bachelor’s degree and <bachelor’s degree based on differences in social advancement and lifetime earnings potential [31]. Home address was geocoded in a subset of participants and categorized using Social Vulnerability Index (SVI), a census tract-level composite indicator variable of neighborhood stressors that incorporates 16 measures of socioeconomic status, household characteristics, racial and ethnic minority status, and housing type and transportation [32].

### Urinary EP measurements

Participants provided one or more urine specimens during pregnancy, which were analyzed for EPs by participating laboratories (Supplementary Table 2). We imputed chemical values measured below the limit of detection (LOD) as the LOD/√2 (Supplementary Table 3) [33]. Urine samples submitted to the different study laboratories were returned with either specific gravity or creatinine values. Every study participant had either a urinary specific gravity or urinary creatinine value reported. Depending on which was reported, a correction was applied to correct for differences in urinary dilution, by multiplying the measurement by the ratio of the creatinine or specific gravity in a reference population to the participant’s observed creatinine or specific gravity, respectively, using the Boeniger method [34], as recently recommended for combining cohorts with different measures of urinary dilution [35]. We considered the following EPs measured widely among participating cohorts and implicated as EDCs: 2,4-dichlorophenol (2,4-DCP), 2,5-dichlorophenol (2,5-DCP), benzophenone 3 (BP-3), bisphenol A (BPA), bisphenol F (BPF), bisphenol S (BPS), methyl paraben (MePb), ethyl paraben (EtPb), propyl paraben (PrPb), and butyl paraben (BuPb). Common routes and sources of exposure are summarized in Supplementary Table 1.

### Data analysis

To estimate associations of racial/ethnic categories and educational level with urinary chemical concentrations, we applied linear mixed regression models with a censored normal distribution, including a random intercept for participants. Urine specimens were analyzed at different laboratories, employing different methods and instruments that had distinct LODs, so LOD values vary across the cohorts as shown in Supplementary Table 3. We used a censored regression model to help address this challenge in pooling the laboratory results from the different cohorts. Such models can accommodate varying left-censored observations lower than the LOD by partitioning the likelihood function into components predicting values lesser and greater than the LOD. Specifically, the model first creates an indicator variable that flags whether a measured value is below or above the LOD. This indicator variable is included in the model to appropriately account for differences in LOD across cohorts and optimization is either based on an expectation maximization algorithm or Gauss-Hermite quadrature [36, 37].

In all of the multivariable models, we adjusted for maternal highest education level, ECHO cohort, gestational age at specimen collection (in weeks), season of specimen collection, maternal age at specimen collection (in years), and maternal pre-pregnancy body mass index (in kg/m^2^) as fixed effects. Covariates were selected based on hypothesized relationships of racial/ethnic identity with urinary chemical concentrations according to the literature using a directed acyclic graph [38, 39] (Supplementary Fig. 3). We did not adjust for year of urine collection as it was colinear to study cohort. To evaluate effect measure modification in the pattern of associations, we stratified the educational level predictor model by racial/ethnic identity. To address the potential impact of neighborhood-level confounding and to disentangle influences of structural socioeconomic disadvantage from self-reported race/ethnicity, we performed sensitivity analyses in which we adjusted for SVI in a subsample of 2117 participants with a geocoded home address. To evaluate the influence of gestational age at urine collection, we performed sensitivity analyses using only second trimester data, which accounted for the majority of urine specimens collected. We also performed a leave-one-cohort-out analysis to assess the influence of individual ECHO cohorts.

We used multiple imputation by chained equations to impute missing covariates and pooled estimates from the imputed data sets using Rubin’s rules. During sensitivity analyses, the list of covariates adjusted in each model varied based on data availability. Stratifying the dataset exclusively to a specific race/ethnicity or educational level resulted in scenarios where certain variables did not exhibit variability and were excluded from the analysis. Furthermore, because of the unbalanced nature of repeated measurements, stratifying the dataset during sensitivity analyses resulted in datasets with one observation per subject or all observations above the LOD for certain strata. We used general linear or linear mixed effects models, respectively, in these scenarios. Statistical significance was defined as a 2-sided *p* < 0.05. We further adjusted the type-1 error rate using a conservative Bonferroni approach for the effective number of tests of each predictor, as 0.05/10 = 0.005 [40]. Statistical analyses were performed using R statistical software, v.4.2.2 (R Foundation for Statistical Computing, Vienna, Austria).

## Results

### Sociodemographic characteristics of the participants

Study participants self-reported Hispanic (41.4%), non-Hispanic Black (12.2%), non-Hispanic Other (9.0%), and non-Hispanic White (36.9%) race and ethnicity (Table 1). Approximately half (46.8%) had completed a bachelor’s degree. The mean gestational age at urine collection was 20.1 weeks, with an interquartile range of 14–26 weeks.

**Table 1:** Distribution of demographic and socioeconomic characteristics among pregnant ECHO study participants (*n* = 4006).

Characteristics	No. (%)
Maternal racial/ethnic identity	
Hispanic	1658 (41.4%)
Non-Hispanic White	1478 (36.9%)
Non-Hispanic Black	490 (12.2%)
Non-Hispanic Asian/Multiple/Other	362 (9.0%)
Missing	18 (0.4%)
Maternal educational attainment	
<Bachelor’s degree	2020 (50.4%)
≥Bachelor’s degree	1874 (46.8%)
Missing	112 (2.8%)
Maternal age at assessment (years)	
Mean (SD)	29.37 (5.69)
Median (IQR)	30 (25, 33)
Range	16 - 48
Missing	<5
Maternal pre-pregnancy BMI (kg/m^2^)	
Mean (SD)	26.64 (6.48)
Median (IQR)	25.1 (22.0, 22.9)
Range	13.2–82.0
Missing	307 (7.7%)
Gestational age at specimen collection (weeks)	
Mean (SD)	20.1 (7.8)
Median (IQR)	20.0 (14.0, 26.0)
Range	0.01–40.00
Missing	0 (0%)

*BMI* body mass index, *ECHO* Environmental influences on Child Health Outcomes, *IQR* interquartile range, *SD* standard deviation.

Includes individuals who have at least one urinary phenol or paraben measurement. In accordance with ECHO’s publication and data use policy, symbols < or > are used to display numbers where there exists a cell size greater than 0 but less than 5, and there is a potential risk of re-identifying participants. Cells with a small size and a few surrounding cells are sufficiently suppressed to prevent back calculation of the exact numbers in the cells with the small size.

### Distributions of urinary EP concentrations

Ten urinary chemicals were measured in participants (Supplementary Table 4). Nine of the 10 EPs were detected in a majority of participants, except for BPF (40.31% > LOD). MePb had the highest median urinary concentration (58.56 µg/L), and BuPb had the lowest (0.16 µg/L). There were moderate to strong positive correlations among Log-transformed urinary EtPb, BuPb, MePb, and PrPb (*r* = 0.34-0.79), and between log-transformed urinary 2,4-DCP and 2,5-DCP (*r* = 0.58) (Supplementary Fig. 4). The distribution of urinary chemicals varied by ECHO cohort (Supplementary Fig. 5).

**Fig. 1:**
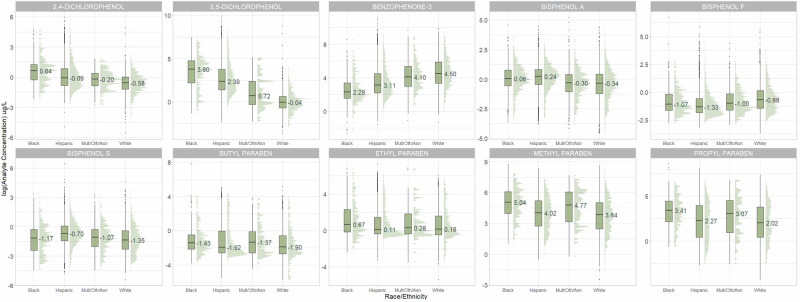
Distributions of natural log-transformed urinary chemical concentrations among pregnant ECHO participants by self-reported racial and ethnic identity. Urinary phenol concentrations (µg/L) corrected for urinary specific gravity or urinary creatinine. Abbreviations: ECHO Environmental influences on Child Health Outcomes, Mult/Oth/Asian non-Hispanic multiple races, “Other,” and Asian.

Boxplots of log-transformed urinary chemical concentrations are shown according to self-reported maternal racial/ethnic identity (Fig. 1). Non-Hispanic Black participants had higher urinary 2,4-DCP, 2,5-DCP, EtPb, MePb, and PrPb concentrations than participants with other racial/ethnic identities. Urinary BPA and BPS concentrations were highest among Hispanic participants, and BP-3 was highest among non-Hispanic White participants.

### Associations between self-reported maternal racial/ethnic identity category and urinary EPs

**Fig. 2:**
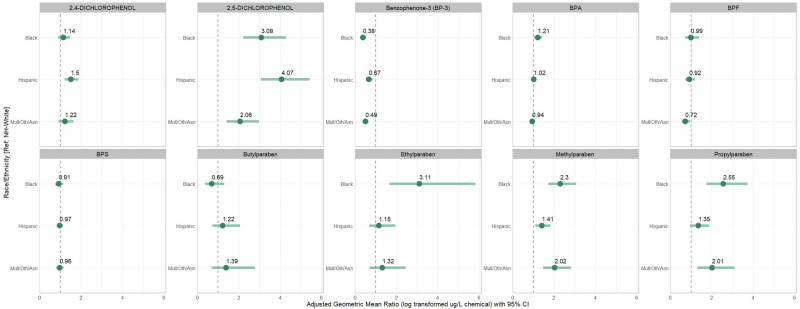
Covariate-adjusted associations between self-reported racial and ethnic identity and urinary chemical concentrations (µg/L) among pregnant ECHO participants. Effect estimates are ratios of geometric means and 95% confidence intervals from individual linear mixed effect censored-response regression models of specific gravity/creatinine-corrected urinary phenol concentrations as outcomes and maternal racial and ethnic identity categories as predictors (non-Hispanic White = reference category), a random intercept on pregnancy to account for multiple urinary measurements and adjusted for maternal age (years), pre-pregnancy body mass index (kg/m^2^), educational level (completed vs. did not complete bachelor’s degree), gestational age at biospecimen collection (weeks), season of biospecimen collection (fall vs. winter vs. spring vs. summer), and ECHO study cohort (11 cohorts). Abbreviations: BPA bisphenol A, BPF bisphenol F, BPS bisphenol S, ECHO Environmental Influences on Child Health Outcomes, Mult/Oth/Asian non-Hispanic multiple races, “Other,” and Asian.

Figure 2 and Supplementary Table 5 show the covariate-adjusted associations between self-reported racial/ethnic identity and urinary chemicals. Relative to non-Hispanic White participants, Hispanic participants had 1.50-fold (95% confidence interval (CI): 1.20–1.87) and 4.07-fold (95% CI: 3.05–5.42) greater urinary 2,4-DCP and 2,5-DCP concentrations, respectively, but a 0.67-fold (95% CI: 0.52–0.85) lower urinary BP-3 level; non-Hispanic Black participants had 3.08-fold (95% CI: 2.22–4.27), 2.30-fold (95% CI: 1.73–3.06), 3.11-fold (95% CI: 1.66–5.82), and 2.55-fold (95% CI: 1.74–3.72) higher urinary 2,5-DCP, MePb, EtPb, and PrPb levels, respectively. Relative to non-Hispanic White participants, non-Hispanic Black participants had 0.38-fold (95% CI: 0.27–0.51) lower urinary BP-3 concentrations; non-Hispanic Other participants had 2.06-fold (95% CI: 1.42–2.99), 2.02-fold (95% CI: 1.46–2.80), and 2.01-fold (95% CI: 1.30–3.11) higher urinary 2,5-DCP, MePb, and PrPb levels, respectively, but a 0.49-fold (95% CI: 0.37–0.65) lower urinary BP-3 level.

The results were similar, but somewhat attenuated, when we adjusted for the SVI in a sensitivity analysis of 2117 participants with a geocoded home address (Supplementary Table 6) and when we limited the analysis to urine specimens collected during the second trimester (Supplementary Table 7). The results of the leave-one-cohort-out analysis were mostly consistent with the main findings (Supplementary Fig. 6). However, exclusion of The Infant Development and Environment Study (TIDES) cohort changed the direction of the effect estimates, with urinary BPA concentrations similar between non-Hispanic Black and non-Hispanic White participants and lower among Hispanic and non-Hispanic Other participants than non-Hispanic White participants. There were also increases in the magnitude of the association of race/ethnic identity with BPF among Hispanic participants and with BPS among Hispanic, non-Hispanic Black, and non-Hispanic Other participants relative to non-Hispanic White participants when excluding the New York University Child Health and Environment Study (NYU-CHES) cohort.

### Associations between maternal educational level and urinary EPs

Table 2 shows the associations between maternal educational level and urinary EPs, adjusted for covariates, according to maternal racial/ethnic identity. In all racial and ethnic groups, participants who had not completed a bachelor’s degree had lower urinary BP-3 than participants who had completed a bachelor’s degree or more, although with statistical significance only for Hispanic (0.68-fold; 95% CI: 0.55–0.84) and non-Hispanic Other (0.44-fold; 95% CI: 0.25–0.77) participants following the Bonferroni adjustment procedure. There was also a consistent pattern of higher urinary BPS and 2,5-DCP among participants who had not completed a bachelor’s degree in all racial and ethnic identity groups, although without statistical significance. Supplementary Table 8 shows a similar pattern of associations between maternal educational level and gestational urinary BP-3, BPS, and 2,5-DCP concentrations in the overall sample.

**Table 2:** Associations between maternal education and urinary chemicals among pregnant ECHO participants by self-reported racial/ethnic identity.

Hispanic	Ratio of GMs	95% CI low	95% CI high	*p*-value	Non-Hispanic White	Ratio of GMs	95% CI low	95% CI high	*p*-value
2,4-dichlorophenol	0.91	0.75	1.10	0.32	2,4-dichlorophenol	0.94	0.78	1.14	0.56
2,5-dichlorophenol	1.06	0.83	1.35	0.64	2,5-dichlorophenol	1.18	0.93	1.49	0.17
Benzophenone-3	**0.68**	**0.55**	**0.84**	**<0.001**	Benzophenone-3	0.73	0.55	0.97	0.03
Bisphenol A	0.99	0.88	1.12	0.90	Bisphenol A	1.16	0.95	1.43	0.14
Bisphenol F	0.98	0.77	1.23	0.83	Bisphenol F	0.99	0.71	1.36	0.94
Bisphenol S	1.16	1.01	1.33	0.04	Bisphenol S	1.12	0.90	1.40	0.32
Butyl Paraben	0.70	0.44	1.10	0.13	Butyl Paraben	1.23	0.73	2.07	0.45
Ethyl Paraben	0.55	0.35	0.87	0.01	Ethyl Paraben	0.83	0.51	1.35	0.46
Methyl Paraben	0.90	0.73	1.10	0.29	Methyl Paraben	1.13	0.86	1.50	0.38
Propyl Paraben	0.83	0.63	1.10	0.19	Propyl Paraben	0.99	0.69	1.42	0.94
**Non-Hispanic Black**	**Ratio of GMs**	**95% CI low**	**95% CI high**	***p*-value**	**Non-Hispanic Other**	**Ratio of GMs**	**95% CI low**	**95% CI high**	***p*-value**
2,4-dichlorophenol	1.52	0.87	2.67	0.14	2,4-dichlorophenol	1.35	0.71	2.57	0.36
2,5-dichlorophenol	1.81	0.93	3.52	0.08	2,5-dichlorophenol	4.80	1.51	15.32	0.01
Benzophenone-3	0.45	0.23	0.87	0.02	Benzophenone-3	**0.44**	**0.25**	**0.77**	**0.004**
Bisphenol A	0.95	0.74	1.21	0.66	Bisphenol A	1.31	0.92	1.86	0.13
Bisphenol F	1.02	0.52	1.97	0.96	Bisphenol F	0.83	0.40	1.72	0.62
Bisphenol S	1.47	0.84	2.60	0.18	Bisphenol S	1.12	0.71	1.76	0.63
Butyl Paraben	0.57	0.20	1.58	0.28	Butyl Paraben	0.43	0.11	1.73	0.24
Ethyl Paraben	0.41	0.13	1.29	0.13	Ethyl Paraben	0.54	0.11	2.63	0.45
Methyl Paraben	0.53	0.28	1.00	0.05	Methyl Paraben	2.02	0.73	5.60	0.17
Propyl Paraben	0.70	0.33	1.51	0.37	Propyl Paraben	3.90	1.15	13.22	0.03

*CI* confidence interval, *ECHO* Environmental influences on Child Health Outcomes, *GM* geometric mean.

Effect estimates are ratios of geometric means and 95% confidence intervals from individual linear mixed effect censored-response regression models of specific gravity/creatinine-corrected urinary phenol and paraben concentrations as outcomes and maternal educational level (<bachelor’s degree vs. ≥bachelor’s degree), a random intercept on pregnancy to account for multiple urine measurements, and adjusted for maternal age (years), pre-pregnancy body mass index (kg/m^2^), gestational age at biospecimen collection (weeks), season of biospecimen collection (fall vs. winter vs. spring vs. summer), and study cohort (11 cohorts). Bold font indicates statistically significant result after correction for multiple comparisons with *p* < 0.005 (i.e., α = 0.05/10 tests).

## Discussion

In this investigation of 4006 pregnant ECHO participants, we found that average urinary EP concentrations differed by self-reported racial/ethnic identity. Non-Hispanic Black and Hispanic participants had greater average urinary concentrations of 2,5-DCP, the primary metabolite of paradichlorobenzene [4], than non-Hispanic White participants. Paradichlorobenzene is used in mothballs, fumigants, and room/toilet deodorizers, allowing the chemical to be inhaled [5]. It is neurotoxic and weakly antiestrogenic in rodents [41], and exposure has been associated with estrogen-sensitive cancers [42]. Urinary MePb, EtPb, and PrPb levels were also higher among non-Hispanic Black than non-Hispanic White participants. These chemicals are weakly estrogenic and used as preservatives in prepared foods and personal care products, allowing them to be ingested and absorbed [8]. Higher gestational exposure to MePb was associated with greater risks of adverse birth outcomes and attention-deficit hyperactivity disorder among offspring [43]. In contrast, average urinary concentrations of BP-3, a UV-filtering chemical absorbed from sunscreens and personal care products, were highest among non-Hispanic White participants. BP-3 has been found to be estrogenic in experimental models, and exposure was associated with adverse reproductive outcomes in human studies [6]. However, we found that the associations did not differ by educational attainment, suggesting that factors other than educational attainment, as a proxy for socioeconomic position, played an important role in racial/ethnic differences. Differential exposure may account in part for racial/ethnic differences in perinatal health outcomes.

### Comparison with previous studies

Pregnant people from across the U.S. with racial and ethnic identities other than non-Hispanic White had higher urinary concentrations of most measured EPs than their non-Hispanic White counterparts. Our results are largely consistent with the results of several previous studies of pregnant people that have also reported racial and ethnic differences in urinary EPs among smaller samples of the U.S. population from limited areas [20–24]. Biomonitoring studies have also described similar racial and ethnic differences in urinary EPs among representative samples of the general U.S. population [19, 44–46]. However, unlike the general U.S. population samples that included people without pregnancy, children, and seniors, our study focused on pregnant people.

Similar to our results, the 2009–2010 U.S. National Children’s Study Vanguard Study (NCS) of 506 pregnant women (some of whom were included in this analysis) showed higher urinary 2,5-DCP levels among non-Hispanic Black than non-Hispanic White participants [20]. Urinary 2,5-DCP levels were similarly lowest among non-Hispanic White participants and those with the highest educational level in the 2009–2014 Healthy Start study of 446 pregnant women from Colorado (some of whom were included in this analysis) [21]. African Americans, a non-Hispanic Black group, had the highest urinary 2,4-DCP and 2,5-DCP levels in the 2006–2008 LIFECODES study of 480 pregnant women from Boston, Massachusetts [22]. These results are consistent with our own findings and with those from a representative sample of U.S. women from 1999–2014, for whom urinary concentrations of 2,4-DCP and 2,5-DCP levels were higher among non-Hispanic Black and Hispanic women than non-Hispanic White women [44]. Similar to the U.S. biomonitoring study, we did not find an association between urinary 2,4-DCP and 2,5-DCP and educational level [44].

In addition, our findings were consistent with results from a 2003–2004 study showing that U.S. non-Hispanic White participants had greater average urinary BP-3 than non-Hispanic Black and Mexican American participants [19]. Pregnant non-Hispanic White women had similarly higher urinary BP-3 concentrations than other racial/ethnic groups in the NCS and Healthy Start studies [20, 21], and BP-3 levels were positively correlated to educational level in the Healthy Start and LIFECODES studies [21, 22]. We also found higher BP-3 levels among pregnant people with more education.

BPA is a plastic monomer used in polycarbonate plastics, epoxy can linings, heat transfer papers, and other consumer goods [7]. BPA levels were similar across different racial/ethnic categories among U.S. women in 1999–2014 [44]. In contrast, urinary levels of BPS, a BPA-replacement chemical, were highest among non-Hispanic Black women, and urinary levels of BPF, another BPA replacement, were highest among non-Hispanic White women from 1999–2016; these differences could not be attributed to income as an indicator of socioeconomic position [44]. Urinary BPS and BPA were similarly highest among non-Hispanic Black U.S. adults from 2007–2016, but there was no significant difference in BPF; concentrations were greatest among those with the lowest education [45]. In contrast, urinary BPA levels were similar among 233 non-Hispanic White, Hispanic, and Other (including Asian, Black, and multiracial) pregnant California women enrolled in the Markers of Autism Risk in Babies–Learning Early Signs (MARBLES) study from 2007–2014, although those with less education had higher urinary BPA levels [23]. We did not find a statistically significant difference in urinary BPA, BPS, or BPF levels between racial/ethnic categories after the Bonferroni adjustment, although our results suggested higher urinary BPA among non-Hispanic Black compared to non-Hispanic White participants. We also did not find associations of BPA, BPF, or BPS with educational level. The differences between our results and those from U.S. biomonitoring data may in part reflect higher intraindividual variabilities in prior studies based on a single urine specimen [47] and different time-activity exposure patterns between pregnant and non-pregnant populations [48].

Our results were similar to those reported in a previous analysis of the Healthy Start Study, in which non-Hispanic Black participants and participants with other racial/ethnic identities had the highest urinary MePb, EtPb, and PrPb levels and non-Hispanic Black participants had the lowest urinary BuPb levels [21]. Higher education was related to higher urinary EtPb and PrPb levels in Healthy Start. Similarly, urinary MePb and PrPb levels were greatest among African American participants, whereas BuPb levels were greatest among White participants in the LIFECODES study [22]. In the Vitamin D Antenatal Asthma Reduction Trial (VDAART), a study of 467 pregnant women from Boston, Massachusetts, maternal plasma MePb and PrPb levels were lowest among non-Hispanic White participants, similar to our findings [24]. Likewise, urinary MePb, EtPb, and PrPb were higher among Hispanic participants and those with other racial/ethnic identities than among White participants in the MARBLES study, and PrPb levels were greater among those with less education [23]. In parallel to our findings among pregnant people, urinary MePb and PrPb concentrations were higher among U.S. non-Hispanic Black, Mexican American, and Other Hispanic participants than among non-Hispanic White participants in 1999–2014, and the differences could not be attributed to socioeconomic position [44].

The results of the current study in a large sample of pregnant people underscore the widespread nature of racial and ethnic differences in urinary EP concentrations, despite decreases in exposure to most EPs in all racial/ethnic groups over time [46].

### Drivers of racial and ethnic differences in urinary EP concentrations

We found differences in urinary EP concentrations between racial/ethnic groups, primarily reflecting higher urinary concentrations among non-Hispanic Black and Hispanic people than among non-Hispanic White people. Yet, we also found that most urinary EPs were similar for participants with different educational levels. These results suggest that the racial/ethnic differences in urinary EPs were similar among participants with different educational levels, which act as a surrogate for socioeconomic position. Personal care products intended for application to the skin, hair, and nails, as well as deodorizers, fragrances, perfumes, and cleansers, are an important source of exposure to parabens and benzophenones [9, 10, 49, 50]. Use of some personal care products differs among White and non-White women [51–54]. While preference and product availability are important, the imposition of Eurocentric beauty standards appears to be a key driver of exposure disparities in non-White populations [9, 51, 55, 56]. Use of products marketed to non-White populations to promote White beauty standards, such as hair relaxers and related haircare products, skin lighteners, and douche/vaginal wash products, can lead to higher EP exposures [12, 57]. Greater use of ethno-targeted beauty products has been associated with increased reproductive health risks [58–60]. Similarly, differences in consumption of processed, packaged, and canned foods leads to different EP exposures [45, 61, 62], and different patterns of product consumption during pregnancy may contribute to the exposure difference [63]. Unfortunately, product selection may be constrained by availability and cost [64], in addition to preference, so the success of individual actions to reduce exposure is likely to be limited; policy-level initiatives are necessary to intervene effectively on the exposure disparity [65]. Resolving the racial and ethnic difference in prenatal EP exposure will require intensive study of the exposure sources to inform greater regulatory attention, and investigation of racial and ethnic differences in perinatal outcomes and child health that can be attributed in part to the different levels of exposure.

### Strengths and limitations

Our sample size of 4006 pregnant people with 7854 urine specimens provided statistical power to detect important differences in urinary EPs among pregnant people with different self-reported racial/ethnic identities. The results of our sensitivity analyses suggested that neighborhood-level confounding was unlikely to bias the results. However, the limited number of participants who identified as non-Hispanic Asian, Hawaiian, American Indian, Alaskan Native, multiple races, and as other racial and ethnic identities precluded analyses as separate groups. A future investigation with oversampling of pregnant people having these racial and ethnic identities is necessary to characterize EP exposure disparities. There were modest differences in effect estimates for urinary BPA, BPF, and BPS when we excluded the TIDES and NYU CHES studies, but most results were also robust to a leave-one-cohort-out analysis.

We measured multiple urinary EPs, including the newer BPA-analog compounds BPF and BPS. However, urinary EPs have short half-lives in vivo. Intraclass correlations ranged from 0.25 for BPS to 0.95 for EtPb in repeated urinary specimens collected at 2 week intervals in Healthy Start [21], suggesting that individual measures may not represent exposure across gestation for some chemicals. Still, we included multiple urinary measurements in the regression models for many participants. The results were also mostly similar in a sensitivity analysis limited to second-trimester urinary specimens, which may in part reflect higher concentrations of some EPs at delivery (24 samples collected at delivery) [66]. Furthermore, there were a large number of samples with BPF values lower than the LOD. We implemented a censored linear mixed effects model to accommodate the uncertainty due to these values. We also included cohort as a fixed effect in regression models to adjust for differences between ECHO cohorts, including using different laboratories to measure EPs [67].

## Conclusions

Our results underscore the disproportionately high levels of exposure to EPs among pregnant racial and ethnic minorities in the U.S. Thus, studies of racial/ethnic differences in perinatal health outcomes should account for differences in chemical exposure.

## Supplementary information

Appendix

Supplementary information

## Data Availability

Select de-identified data from the ECHO Program are available through NICHD’s Data and Specimen Hub (DASH). Information on study data not available on DASH, such as some Indigenous datasets, can be found on the ECHO study DASH webpage.
