# Supplementary material for: Racial and ethnic differences in prenatal exposure to environmental phenols and parabens in the ECHO Cohort
Source: J Expo Sci Environ Epidemiol. 2025 Feb 15;35(6):992–1002. doi: 10.1038/s41370-025-00750-w (PMC12583149; doi:10.1038/s41370-025-00750-w)
Supplement: Supplementary file 2 — Supplementary information [file 41370_2025_750_MOESM2_ESM.pdf]

Supplementary Tables and Figures

Supplementary Table 1. Routes and sources of exposure to environmental phenols and parabens measured in this analysis

Supplementary Table 2. Summary of ECHO cohorts included in the final analytical sample

Supplementary Table 3. Average LOD (µg/L) by cohort and chemical

Supplementary Table 4. Distribution of urinary chemical concentrations (µg/L) across all available measurements

Supplementary Table 5. Covariate-adjusted associations (95% confidence intervals) between self-reported racial/ethnic identity category and urinary chemical concentrations among pregnant ECHO participants (n=4006)

Supplementary Table 6. Covariate-adjusted associations (95% confidence intervals) between self-reported racial/ethnic identity category and urinary chemical concentrations among pregnant ECHO participants, also adjusted for the social vulnerability index (n=2117)

Supplementary Table 7. Covariate-adjusted associations (95% confidence intervals) between self-reported racial/ethnic identity category and 2nd trimester urinary chemical concentrations among pregnant ECHO participants (n=2998)

Supplementary Table 8. Covariate-adjusted associations (95% confidence intervals) between participants with < bachelor's degree vs. ≥ bachelor's degree and urinary chemical concentrations among pregnant ECHO participants (n=4006)

Supplementary Figure 1. Flow chart of the analytic sample

Supplementary Figure 2. Study sites of the ECHO cohorts included in the current analysis

Supplementary Figure 3. Directed acyclic graph describing the causal relationships hypothesized among variables in ECHO

Supplementary Figure 4. Correlations between urinary chemical concentrations among pregnant ECHO participants

Supplementary Figure 5. Distributions of natural log-transformed urinary chemical concentrations among pregnant ECHO participants by individual ECHO cohort

Supplementary Figure 6. Covariate-adjusted associations (ratios of geometric means and 95% confidence intervals) between self-reported racial/ethnic identity category and urinary chemicals in pregnant ECHO participants, excluding one cohort at a time (n=4006)

Supplementary Table 1. Routes and sources of exposure to environmental phenols and parabens measured in this analysis

Chemical	Abbreviation	Routes of exposure	Sources of exposure
2,4-dichlorophenol	2,4-DCP	Inhalation	Dyes, pharmaceutical/agricultural products, moth balls and repellents, space deodorants [1,2].
2,5-dichlorophenol	2,5-DCP	Inhalation	Moth balls and repellents, room/toilet deodorizers, pesticides [1,2].
Benzophenone 3 (oxybenzone)	BP-3	Absorption	Sunscreens, personal care products [3].
Bisphenol A	BPA	Ingestion, absorption, injection	Food and beverage packaging, medical equipment, adhesives, building materials, paper coatings [4].
Bisphenol F	BPF	Ingestion	Clear plastics, canned foods and beverages [4].
Bisphenol S	BPS	Ingestion	Canned foods, plastic food wraps [4].
Methyl paraben	MePb	Absorption, ingestion	Packaged foods, personal care products cosmetics, deodorants, hair care products [5].
Ethyl paraben	EtPb	Absorption, ingestion	Packaged foods, personal care products [5].
Propylparaben	PrPb	Absorption, ingestion	Packaged foods, pharmaceuticals, personal care products [5].
Butyl paraben	BuPb	Absorption, ingestion	Packaged foods, pharmaceuticals, personal care products [5].

Supplementary Table 2. Summary of ECHO cohorts included in the final analytical sample

ECHO cohort	No. of unique participants	Total specimens	Total measurements	Location	Years urine collected	Trimester urine collected	Laboratory & analysis method used	Chemicals measured
ECHO Puerto Rico	777	1737	17627	Puerto Rico	2012–2020	1, 2, 3	CDC Lab using HPLC-MS/MS	2,4-DCP, 2,5-DCP, BP-3, BPA, BPF, BPS, MePb, EtPb, PrPb, BuPb
Healthy Start Study	446	446	4013	Colorado	2011–2014	2, 3	CDC Lab using LC-isotope dilution MS/MS	2,4-DCP, 2,5-DCP, BP-3, BPA, BPS, MePb, EtPb, PrPb, BuPb
Atlanta ECHO Cohort of Emory University	150	349	699	Georgia	2016–2018	1, 2, 3	CHEAR Lab at Emory University using LC-ESI-MS/MS & HPLC-MS/MS	BP-3, BPA, BPF, BPS, MePb, EtPb, PrPb, BuPb
Pregnancy and EnvironmenT And Lifestyle Study (PETALS)	367	731	2228	Northern California	2013–2017	1, 2, 3	California Public Health Environmental Health Lab & CDC Lab using LC/MS/MS	BP-3, BPA, BPF, BPS
New Hampshire Birth Cohort Study (NHBCS)	108	136	1320	New Hampshire	2017–2019	1, 2, 3	HHEAR Lab at Wadsworth Center-NYSDOH using HPLC-MS/MS	2,4-DCP, 2,5-DCP, BP-3, BPA, BPF, BPS, MePb, EtPb, PrPb, BuPb,

Archive for Research in Child Health (ARCH)	172	172	623	Michigan	2008–2016	1, 2, 3	Wadsworth Center-NYSDOH Lab using LC-MS/MS	MePb, EtPb, PrPb, BuPb
Illinois Kids Development Studies (IKIDS)	410	410	4070	Illinois	2014–2019	1, 2, 3	Wadsworth Center-NYSDOH Lab using HPLC-MS/MS	2,4-DCP, 2,5-DCP, BP-3, BPA, BPF, BPS, MePb, EtPb, PrPb, BuPb,
Utah Children's Project	33	45	450	Utah	2011–2012	1, 2, 3	CHEAR Lab at Wadsworth Center-NYSDOH using HPLC-MS/MS	2,4-DCP, 2,5-DCP, BP-3, BPA, BPF, BPS, MePb, EtPb, PrPb, BuPb
New York University Child Health and the Environment Study (NYU CHES)	534	1593	4779	New York City	2016–2018	1, 2, 3	Wadsworth Center-NYSDOH Lab using HPLC-MS/MS	BPA, BPF, BPS
The Infant Development and the Environment Study (TIDES)	643	1869	5604	California, Minnesota, New York, Washington	2009–2013	1, 2, 3	Wadsworth Center-NYSDOH Lab using HPLC-MS/MS	BPA, BPF, BPS
Mothers and Newborns Study	366	366	2541	New York City	1999–2006	3	CDC Lab using HPLC-ESI-MS	2,4-DCP, 3,5-DCP, BP-3, BPA, MePb, PrPb, BuPb

Abbreviations: BPA, bisphenol A; BPF, bisphenol F; BPS, bisphenol S; BP-3, benzophenone-3; BuPb, butyl paraben; CDC, U.S. Centers for Disease Control and Prevention; CHEAR, Child Health Exposure Analysis Resource; ECHO, Environmental Influences on Child Health Outcomes; ESI, electrospray ionization; EtPb, ethyl paraben; HHEAR, Human Health Exposure Analysis Resource; HPLC, high performance-liquid chromatography; LC, liquid chromatography; MePb, methyl paraben; MS, mass spectrometry; NYSDOH, New York State Department of Health; PrPb, propyl paraben; 2,4-DCP, 2,4-dichlorophenol; 2,5-DCP, 2,5-dichlorophenol

Supplementary Table 3. Average LOD (μg/L) by cohort and chemical

ECHO cohort	2,4-DCP	2,5-DCP	BP-3	BPA	BPF	BPS	MePb	EtPb	PrPb	BuPb
ECHO Puerto Rico	0.15	0.15	0.2333	0.1925	0.2	0.075	0.525	0.505	0.15	0.11667
Healthy Start Study	0.1	0.1	0.2	0.1	-	0.1	1	1	0.1	0.1
Atlanta ECHO Cohort of Emory University	-	-	0.1	0.235	0.35	0.05	0.525	0.255	0.575	0.275
Pregnancy and EnvironmenT And Lifestyle Study (PETALS)	-	-	0.7	0.2	0.2	0.1	-	-	-	-
New Hampshire Birth Cohort Study (NHBCS)	0.04	0.054	0.45	0.0515	0.131	0.032	0.066	0.018	0.089	0.03
Archive for Research in Child Health (ARCH)	-	-	-	-	-	-	0.3	0.06	0.3	0.06
Illinois Kids Development Studies (IKIDS)	0.1	0.1	0.25	0.135	0.2	0.075	0.525	0.505	0.125	0.075
Utah Children's Project	0.01	0.05	0.05	0.1	0.1	0.01	0.5	0.1	0.01	0.01
New York University Child Health and the Environment Study (NYU CHES)	-	-	-	0.15	0.359	0.078	-	-	-	-
The Infant Development and the Environment Study (TIDES)	-	-	-	0.15	0.359	0.078	-	-	-	-
Mothers and Newborns Study	0.2	0.2	0.4	0.4	-	-	1	-	0.2	0.2

Abbreviations: BPA, bisphenol A; BPF, bisphenol F; BPS, bisphenol S; BP-3, benzophenone-3; BuPb, butyl paraben; ECHO, Environmental Influences on Child Health Outcomes; EtPb, ethyl paraben; LOD, limit of detection; MePb, methyl paraben; PrPb, propyl paraben; 2,4-DCP, 2,4-dichlorophenol; 2,5-DCP, 2,5-dichlorophenol

Supplementary Table 4. Distribution of urinary chemical concentrations (μg/L) across all available measurements

Urinary chemical	Summary	Total	Urinary chemical	Summary	Total
2,4-dichlorophenol	No. of measurements	3084	Bisphenol S	# of measurements	6623
	Median (IQR)	0.82 (0.41, 1.98)		Median (IQR)	0.38 (0.16, 0.87)
	Min - Max	0.004 - 387.20		Min - Max	0.004 - 624
	% > LOD	97.96		% > LOD	77.65
	Years included	1999 - 2020		Years included	2009 - 2020
2,5-dichlorophenol	No. of measurements	3084	Methyl Paraben	# of measurements	3374
	Median (IQR)	6.33 (1.88, 31.03)		Median (IQR)	58.56 (15.58, 190.37)
	Min - Max	0.03 - 17720.21		Min - Max	0.08 - 6628.12
	% > LOD	98.93		% > LOD	99.79
	Years included	1999 - 2020		Years included	1999 - 2020
Benzophenone-3	No. of measurements	3889	Ethyl Paraben	# of measurements	2994
	Median (IQR)	31.53 (1.63, 143.74)		Median (IQR)	1.17 (0.67, 4.76)
	Min - Max	0.072 - 75393		Min - Max	0.004 - 1515.8
	% > LOD	99.56		% > LOD	52.67
	Years included	1999 - 2020		Years included	2008 - 2020
Bisphenol A	No. of measurements	7682	Propyl Paraben	# of measurements	3364
	Median (IQR)	0.99 (0.46, 1.98)		Median (IQR)	10.49 (1.73, 54.55)
	Min - Max	0.01 - 189.685		Min - Max	0.014 - 5396.38
	% > LOD	80.27		% > LOD	99.17
	Years included	1999 - 2020		Years included	1999 - 2020
Bisphenol F	No. of measurements	6199	Butyl Paraben	# of measurements	3301
	Median (IQR)	0.35 (0.18, 0.82)		Median (IQR)	0.16 (0.08, 0.79)
	Min - Max	0.025 - 844.803		Min - Max	0.003 - 2441.68
	% > LOD	40.31		% > LOD	53.98
	Years included	2009 - 2020		Years included	1999 - 2020

Abbreviations: IQR, interquartile range; LOD, limit of detection; Max., maximum measured value; Min., minimum measured value

Supplementary Table 5. Covariate-adjusted associations (95% confidence intervals) between self-reported racial/ethnic identity category and urinary chemical concentrations among pregnant ECHO participants (n=4006)

Category	Ratio of GMs	95% CI low	95% CI high	p-value	Ratio of GMs	95% CI low	95% CI high	p-value
		2,4-dichlorophenol				Bisphenol S		
Hispanic	**1.50**	**1.20**	**1.87**	**<0.001**	0.97	0.83	1.13	0.68
NHB	1.14	0.89	1.47	0.29	0.91	0.75	1.12	0.39
NHO	1.22	0.92	1.62	0.17	0.96	0.81	1.14	0.64
		2,5-dichlorophenol				Methyl Paraben		
Hispanic	**4.07**	**3.05**	**5.42**	**<0.001**	1.41	1.10	1.81	0.01
NHB	**3.08**	**2.22**	**4.27**	**<0.001**	**2.30**	**1.73**	**3.06**	**<0.001**
NHO	**2.06**	**1.42**	**2.99**	**<0.001**	**2.02**	**1.46**	**2.80**	**<0.001**
		Benzophenone-3				Ethyl Paraben		
Hispanic	**0.67**	**0.52**	**0.85**	**0.001**	1.15	0.68	1.94	0.60
NHB	**0.38**	**0.27**	**0.51**	**<0.001**	**3.11**	**1.66**	**5.82**	**<0.001**
NHO	**0.49**	**0.37**	**0.65**	**<0.001**	1.32	0.71	2.44	0.38
		Bisphenol A				Propyl Paraben		
Hispanic	1.02	0.90	1.15	0.76	1.35	0.96	1.88	0.08
NHB	1.21	1.03	1.42	0.02	**2.55**	**1.74**	**3.72**	**<0.001**
NHO	0.94	0.82	1.08	0.39	**2.01**	**1.30**	**3.11**	**0.002**
		Bisphenol F				Butyl Paraben		
Hispanic	0.92	0.71	1.19	0.52	1.22	0.72	2.06	0.47
NHB	0.99	0.72	1.37	0.96	0.69	0.37	1.30	0.25
NHO	0.72	0.55	0.95	0.02	1.39	0.69	2.78	0.36

Individual linear mixed effect censored-response regression models of specific gravity/creatinine-corrected urinary chemical concentrations as outcomes and maternal racial/ethnic identities as predictors (non-Hispanic White = reference category), a random intercept on pregnancy to account for multiple urine measurements, and adjusted for maternal age (years), pre-pregnancy body mass index (kg/m^2), educational level (completed vs. did not complete bachelor's degree), gestational age at biospecimen collection (weeks), season of biospecimen collection (fall vs. winter vs. spring vs. summer), and study cohort (11 cohorts). Bold font indicates statistically significant result after correction for multiple comparisons with p-value < 0.005 (i.e., $\alpha = 0.05/10$ tests)

Abbreviations: CI, confidence interval; ECHO, Environmental Influences on Child Health Outcomes; GM, geometric mean; NHB, non-Hispanic Black; NHO, non-Hispanic Asian, multiple races, and "other"; NHW, non-Hispanic White

Supplementary Table 6. Covariate-adjusted associations (95% confidence intervals) between self-reported racial/ethnic identity category and urinary chemical concentrations among pregnant ECHO participants, also adjusted for the social vulnerability index (n=2117)

Category	Ratio of GMs	95% CI low	95% CI high	p-value	Ratio of GMs	95% CI low	95% CI high	p-value
		2,4-dichlorophenol[a]				Bisphenol S[a]		
Hispanic	1.39	1.03	1.86	0.03	0.77	0.60	0.99	0.04
NHB	1.02	0.72	1.43	0.92	0.85	0.61	1.18	0.33
NHO	1.08	0.75	1.57	0.68	0.85	0.66	1.09	0.21
		2,5-dichlorophenol[a]				Methyl Paraben[a]		
Hispanic	**2.93**	**2.01**	**4.26**	**<0.001**	1.16	0.75	1.81	0.50
NHB	**2.12**	**1.37**	**3.30**	**0.001**	1.74	1.05	2.88	0.03
NHO	1.35	0.83	2.18	0.22	1.70	0.97	2.98	0.07
		Benzophenone-3[a]				Ethyl Paraben[a]		
Hispanic	0.73	0.51	1.05	0.09	0.88	0.46	1.67	0.70
NHB	**0.47**	**0.30**	**0.75**	**0.001**	2.14	0.99	4.61	0.05
NHO	0.64	0.44	0.95	0.03	1.10	0.53	2.28	0.80
		Bisphenol A[a]				Propyl Paraben[a]		
Hispanic	1.08	0.89	1.32	0.43	1.17	0.75	1.81	0.50
NHB	1.19	0.92	1.54	0.18	1.74	1.05	2.88	0.03
NHO	0.93	0.76	1.14	0.47	1.70	0.97	2.98	0.07
		Bisphenol F[b]				Butyl Paraben[a]		
Hispanic	1.10	0.58	2.12	0.77	1.42	0.76	2.63	0.27
NHB	1.53	0.55	4.28	0.42	0.83	0.40	1.72	0.62
NHO	0.69	0.35	1.33	0.26	1.55	0.71	3.38	0.27

Individual linear mixed effect censored-response regression models of specific gravity/creatinine-corrected urinary environmental chemical concentrations as outcomes and maternal racial/ethnic identities as predictors (non-Hispanic White = reference category), a random intercept on pregnancy to account for multiple urine measurements, and adjusted for maternal age at pregnancy (years), pre-pregnancy body mass index (kg/m^2), educational level (completed vs. did not complete bachelor's degree), gestational age at biospecimen collection (weeks), season of biospecimen collection (fall vs. winter vs. spring vs. summer), study cohort (11 cohorts), and social vulnerability index. Bold font indicates statistically significant result after correction for multiple comparisons with p-value < 0.005 (i.e., α = 0.05/10 tests)

[a]Analysis excluded ECHO Puerto Rico cohort participants; [b]Analysis excluded ECHO Puerto Rico, Healthy Start, Atlanta ECHO cohort, Pregnancy and EnvironmenT And Lifestyle Study (PETALS), New Hampshire Birth Cohort Study, Illinois Kids Development Study (IKIDS), Utah Children's Project, and New York University Child Health and the Environment (NYU CHES) cohort participants

Abbreviations: CI, confidence interval; ECHO, Environmental Influences on Child Health Outcomes; GM, geometric mean; NHB, non-Hispanic Black; NHO, non-Hispanic Asian, multiple races, and "other"; NHW, non-Hispanic White

Supplementary Table 7. Covariate-adjusted associations (95% confidence intervals) between self-reported racial/ethnic identity category and 2nd trimester urinary chemical concentrations among pregnant ECHO participants (n=2998)

Category	Ratio of GMs	95% CI low	95% CI high	p-value	Ratio of GMs	95% CI low	95% CI high	p-value
		2,4-dichlorophenol				Bisphenol S		
Hispanic	1.41	1.01	1.97	0.05	0.96	0.80	1.14	0.62
NHB	1.31	0.84	2.04	0.23	1.03	0.81	1.32	0.78
NHO	1.19	0.84	1.68	0.33	0.98	0.81	1.19	0.86
		2,5-dichlorophenol				Methyl Paraben		
Hispanic	**3.44**	**2.18**	**5.43**	**<0.001**	1.40	0.92	2.12	0.11
NHB	**3.89**	**2.15**	**7.04**	**<0.001**	**4.52**	**2.65**	**7.71**	**<0.001**
NHO	**2.05**	**1.29**	**3.27**	**0.002**	1.49	0.97	2.29	0.07
		Benzophenone-3				Ethyl Paraben		
Hispanic	0.71	0.51	0.99	0.04	1.34	0.60	3.03	0.48
NHB	**0.27**	**0.16**	**0.45**	**<0.001**	**4.31**	**1.59**	**11.73**	**<0.001**
NHO	**0.46**	**0.33**	**0.64**	**<0.001**	0.97	0.41	2.29	0.95
		Bisphenol A				Propyl Paraben		
Hispanic	1.02	0.87	1.20	0.78	1.42	0.82	2.48	0.21
NHB	1.14	0.90	1.43	0.27	**4.39**	**2.15**	**8.95**	**<0.001**
NHO	0.97	0.82	1.16	0.74	1.63	0.91	2.92	0.10
		Bisphenol F				Butyl Paraben		
Hispanic	0.79	0.56	1.11	0.18	0.95	0.38	2.35	0.91
NHB	0.74	0.47	1.17	0.20	0.56	0.16	1.93	0.36
NHO	0.63	0.44	0.91	0.01	1.00	0.38	2.61	1.00

Individual linear mixed effect censored-response regression models of specific gravity/creatinine-corrected urinary chemical concentrations as outcomes and self-reported racial/ethnic identity categories as predictors (non-Hispanic White = reference category), a random intercept on pregnancy to account for multiple urine measurements, and adjusted for maternal age (years), pre-pregnancy body mass index (kg/m^2), educational level (completed vs. did not complete bachelor's degree), gestational age at biospecimen collection (weeks), season of biospecimen collection (fall vs. winter vs. spring vs. summer), and study cohort (11 cohorts). Bold font indicates statistically significant result after correction for multiple comparisons with p-value < 0.005 (i.e., α = 0.05/10 tests)

Abbreviations: CI, confidence interval; ECHO, Environmental Influences on Child Health Outcomes; GM, geometric mean; NHB, non-Hispanic Black; NHO, non-Hispanic Asian, multiple races, and "other"; NHW, non-Hispanic White

Supplementary Table 8. Covariate-adjusted associations (95% confidence intervals) between participants with < bachelor's degree vs. ≥ bachelor's degree and urinary chemical concentrations among pregnant ECHO participants (n=4006)

Urinary Chemical	Ratio of GMs	95% CI low	95% CI high	p-value
2,4-dichlorophenol	0.92	0.81	1.05	0.24
2,5-dichlorophenol	1.16	0.98	1.38	0.08
Benzophenone-3	**0.64**	**0.54**	**0.75**	**<0.001**
Bisphenol A	1.04	0.95	1.14	0.43
Bisphenol F	0.95	0.80	1.13	0.57
Bisphenol S	1.15	1.03	1.29	0.02
Methyl Paraben	0.95	0.81	1.11	0.52
Ethyl Paraben	0.64	0.47	0.87	0.01
Propyl Paraben	0.90	0.73	1.10	0.30
Propyl Paraben	0.90	0.73	1.10	0.30
Butyl Paraben	0.78	0.57	1.08	0.14

Individual linear mixed effect censored-response regression models of specific gravity/creatinine-corrected urinary chemical concentrations as outcomes and maternal educational level (≥ bachelor's degree = reference category) as predictor, a random intercept on pregnancy to account for multiple urine measurements, and adjusted for by self-reported racial/ethnic identity category, maternal age (years), maternal pre-pregnancy body mass index (kg/m^2), gestational age at specimen collection (weeks), season of specimen collection (fall vs. winter vs. spring vs. summer), and study cohort (11 cohorts). Bold font indicates statistically significant result after correction for multiple comparisons with p-value < 0.005 (i.e., $\alpha = 0.05/10$ tests)

Abbreviations: CI, confidence interval; ECHO, Environmental Influences on Child Health Outcomes; GM, geometric mean

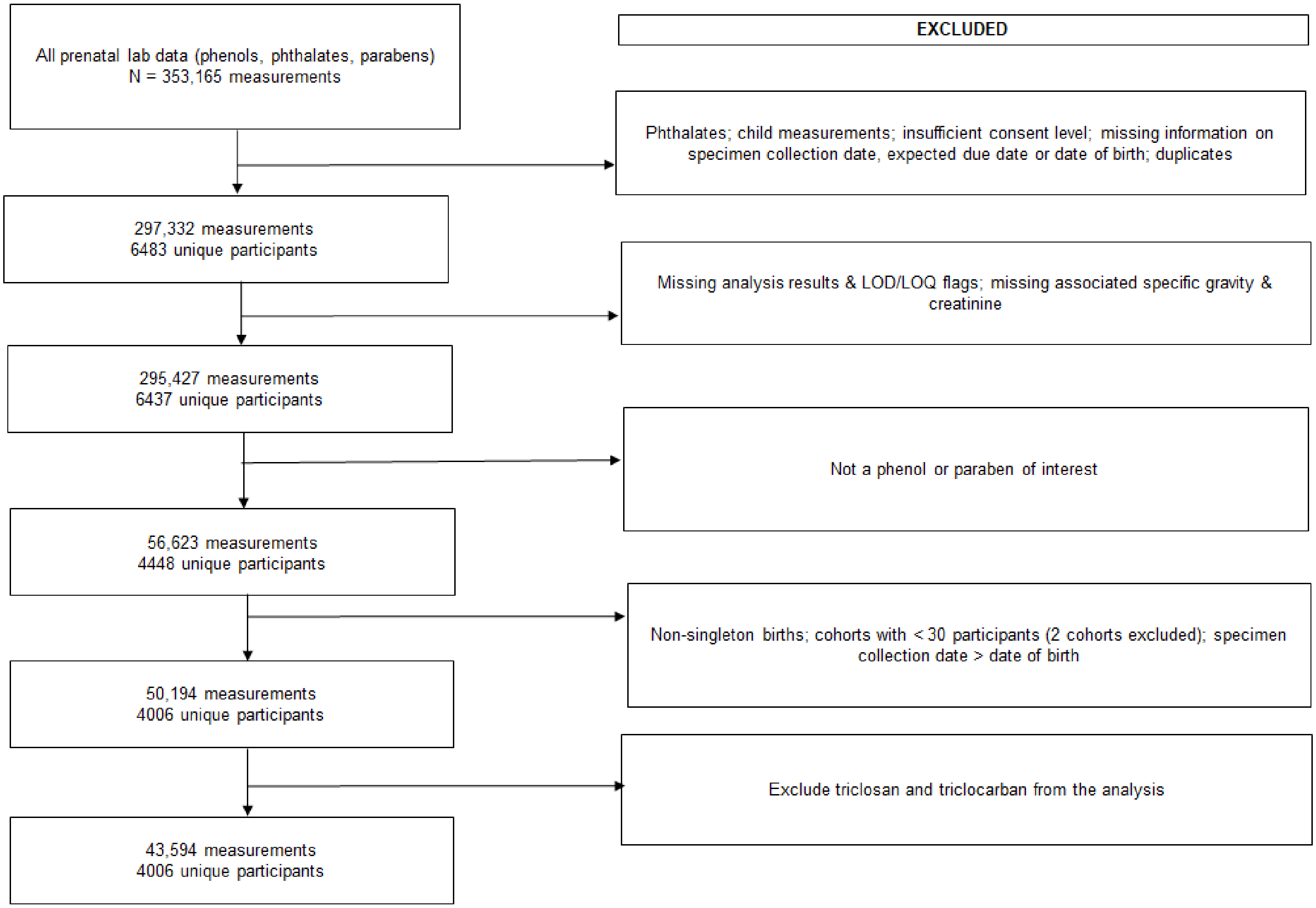

Supplementary Figure 1. Flow chart of the analytic sample

Abbreviations: LOD, limit of detection; LOQ, limit of quantitation

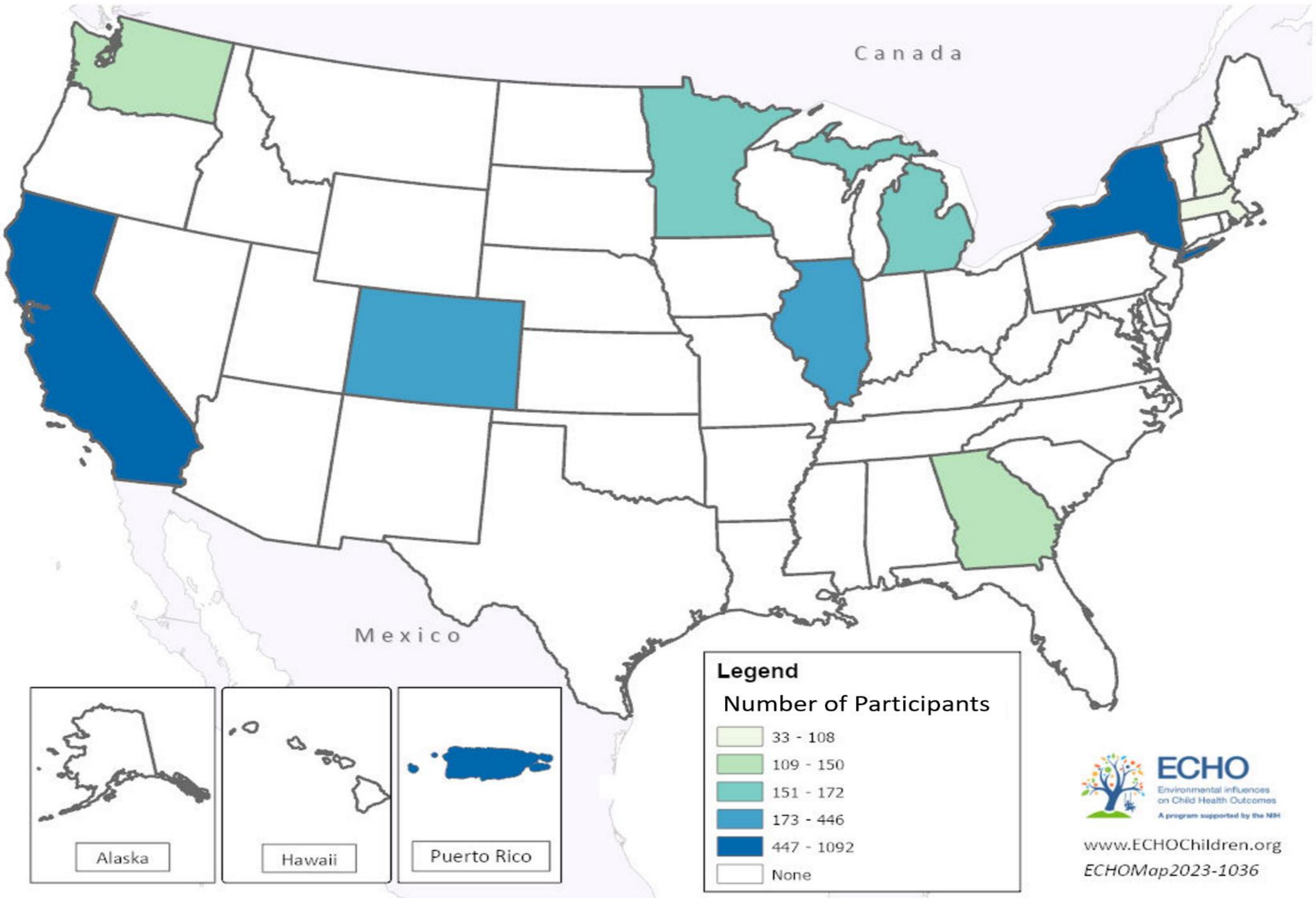

Supplementary Figure 2. Study sites of the ECHO cohorts included in the current analysis

Abbreviations: ECHO, Environmental Influences on Child Health Outcomes

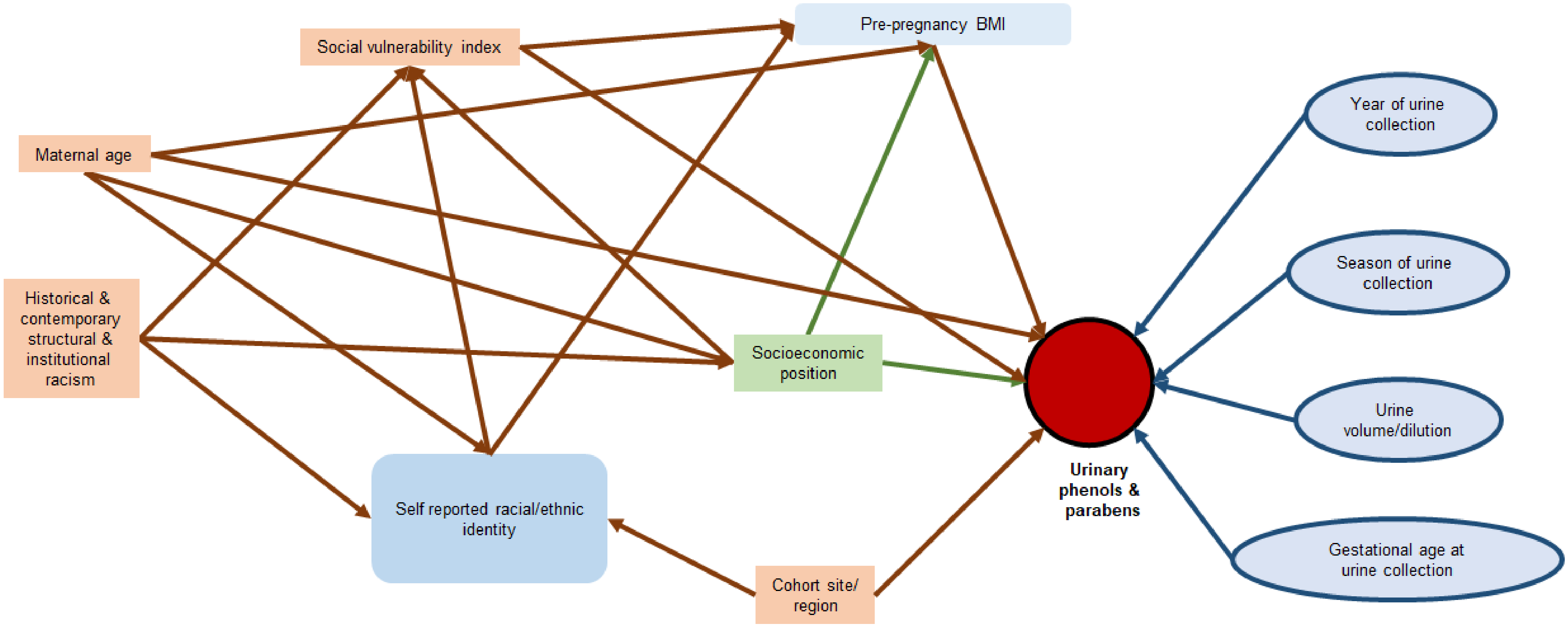

Supplementary Figure 3. Directed acyclic graph describing the causal relationships hypothesized among variables in ECHO

Abbreviations: BMI, body mass index; ECHO, Environmental Influences on Child Health Outcomes

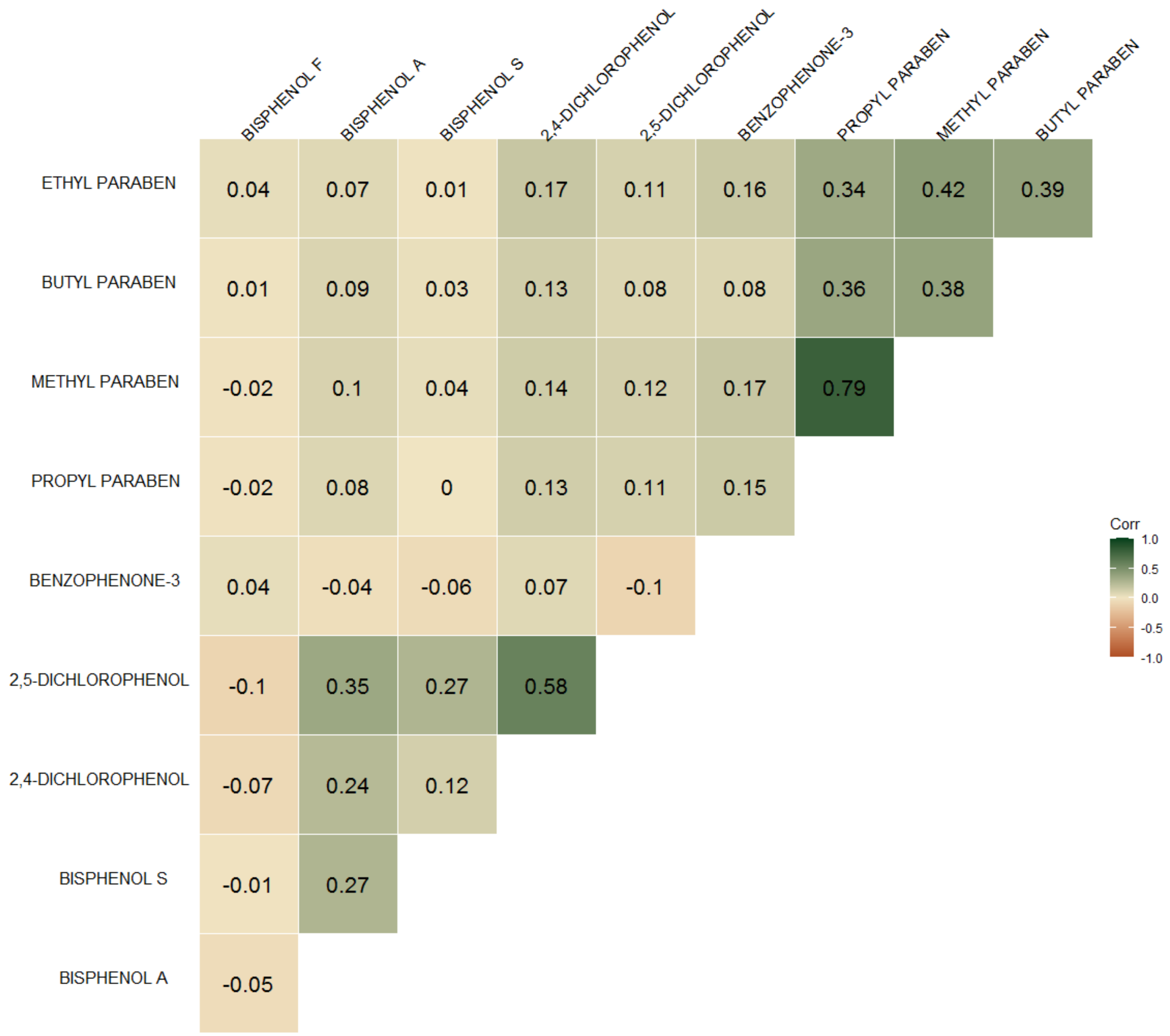

Supplementary Figure 4. Correlations between urinary chemical concentrations among pregnant ECHO participants

Urinary phenol concentrations corrected for urinary specific gravity or urinary creatinine

Abbreviations: ECHO, Environmental Influences on Child Health Outcomes

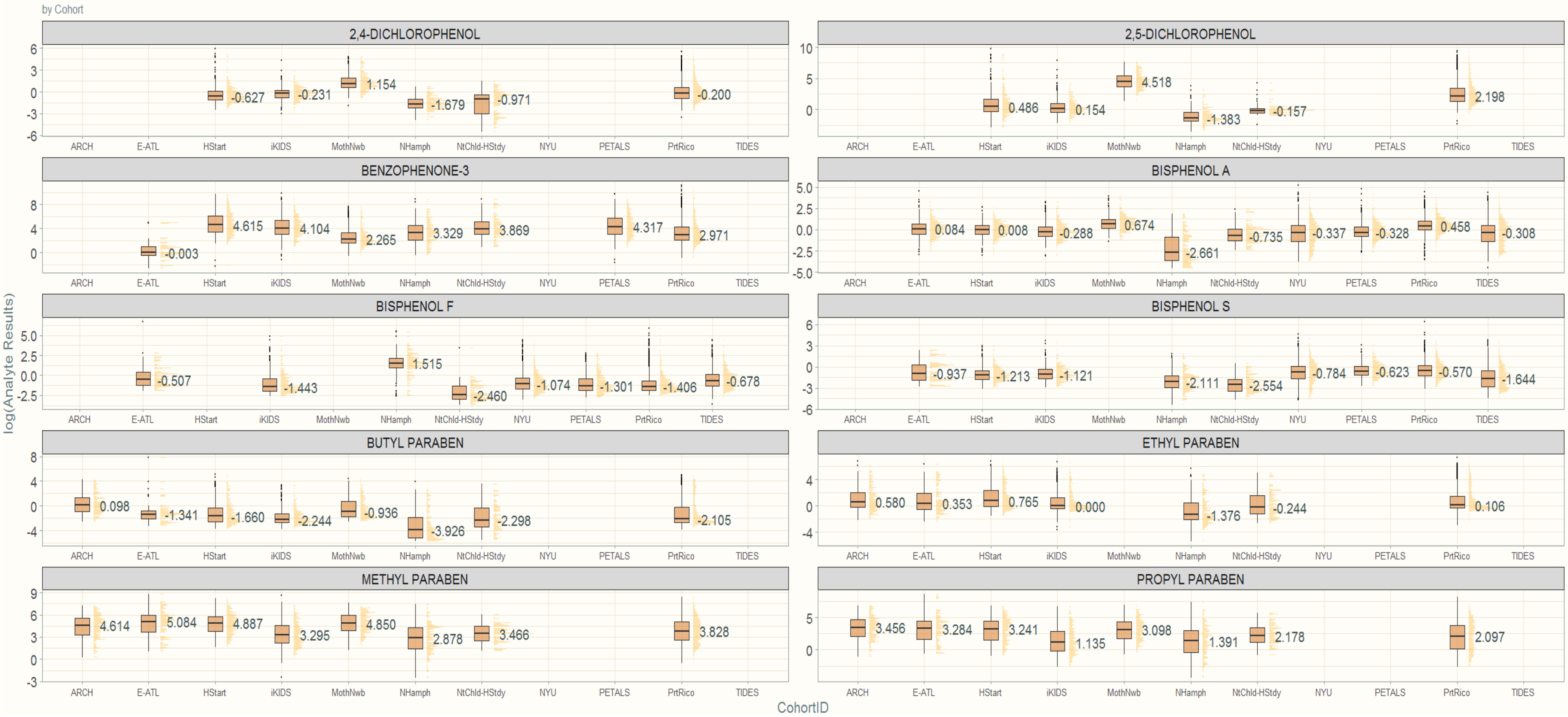

Supplementary Figure 5. Distributions of natural log-transformed urinary chemical concentrations (μg/L) among pregnant ECHO participants by individual ECHO cohort

Urinary phenol concentrations corrected for urinary specific gravity or urinary creatinine

Abbreviations: ARCH, Archive for Research on Child Health cohort; E-ATL, Atlanta ECHO Cohort of Emory University; ECHO, Environmental Influences on Child Health Outcomes; HStart, Healthy Start; IKIDS, Illinois Kids Development Study; MothNwb, Mothers and Newborns; NHamp, New Hampshire Birth Cohort Study; NtChld-HStudy, Utah Children's Project; NYU, New York University Child Health and Environment Study; PETALS, Pregnancy Environment and Lifestyle Study; PrtRico, ECHO Puerto Rico cohort; TIDES, The Infant Development and Environment Study

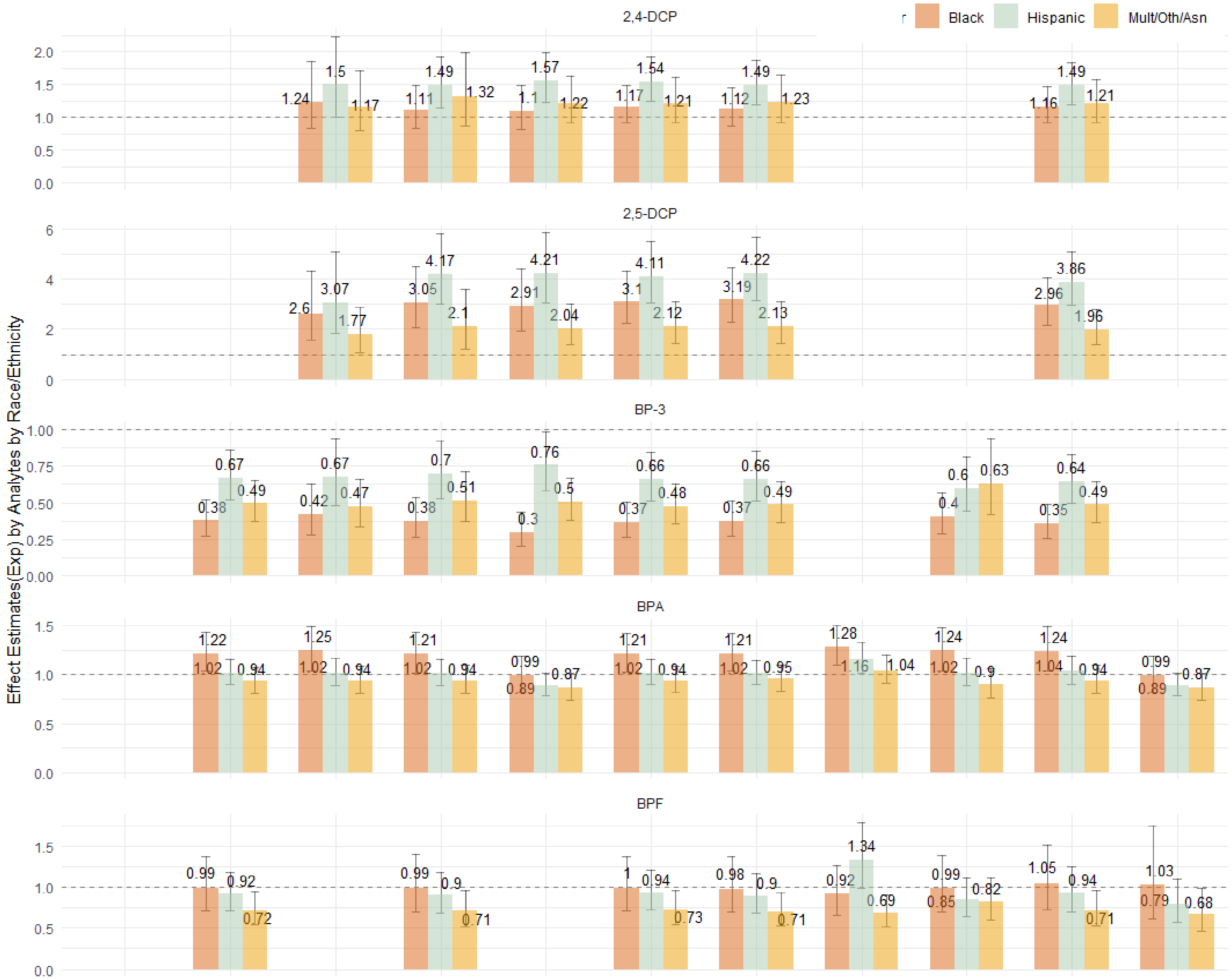
Black
Hispanic
Mult/Oth/Asn
2,4-DCP
2,5-DCP
BP-3
BPA
BPF
Effect Estimates(Exp) by Analytes by Race/Ethnicity

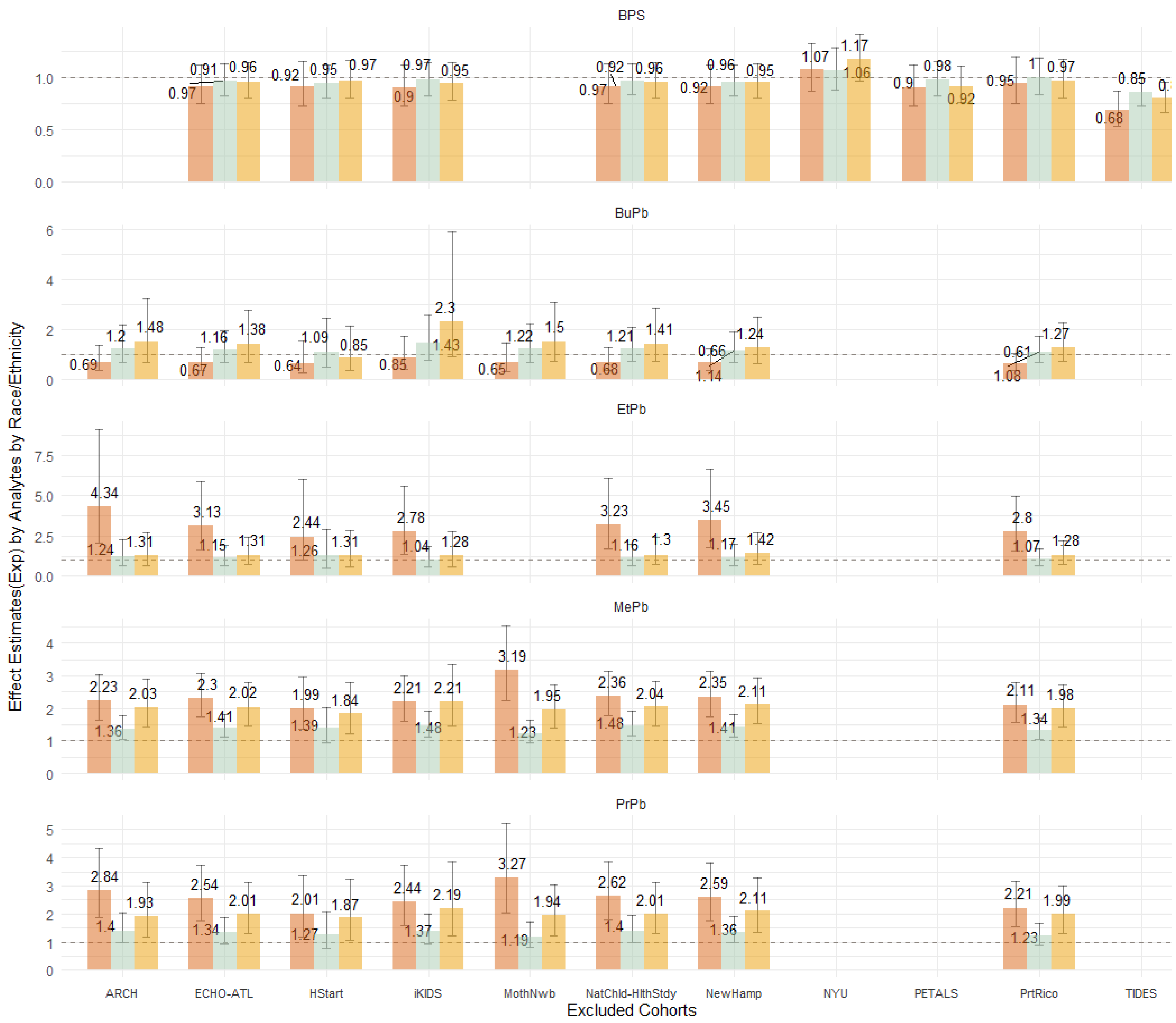
BPS
BuPb
EtPb
MePb
PrPb
Effect Estimates(Exp) by Analytes by Race/Ethnicity
ARCH
ECHO-ATL
HStart
iKIDS
MothNwb
NatChld-HlthStdy
NewHamp
NYU
PETALS
PrtRico
TIDES
Excluded Cohorts

Supplementary Figure 6. Covariate-adjusted associations (ratios of geometric means and 95% confidence intervals) between self-reported racial/ethnic identity category and urinary chemicals in pregnant ECHO participants, excluding one cohort at a time (n=4006)

[a]Individual linear mixed effect censored-response regression models of specific gravity/creatinine-corrected urinary chemical concentrations as outcomes and maternal racial/ethnic identities as predictors (non-Hispanic White = reference category), a random intercept on pregnancy to account for multiple urine measurements, and adjusted for maternal age (years), pre-pregnancy body mass index (kg/n^2), educational level (completed vs. did not complete bachelor's degree), gestational age at biospecimen collection (weeks), season of biospecimen collection (fall vs. winter vs. spring vs. summer), and study cohort (10 cohorts), excluding one study cohort at a time (excluded cohort listed on X axis)

Abbreviations: ARCH, Archive for Research on Child Health; BPA, bisphenol A; BPF, bisphenol F; BPS, bisphenol S; BP-3, benzophenone-3; BuPb, butyl paraben; E-ATL, Atlanta ECHO Cohort of Emory University; ECHO, Environmental Influences on Child Health Outcomes; EtPb, ethyl paraben; HStart, Healthy Start; IKIDS, Illinois Kids Development Study; MePb, methyl paraben; MothNwb, Mothers and Newborns; NHamp, New Hampshire Birth Cohort Study; NtChld-HStudy, Utah Children's Project; NYU, New York University Child Health and Environment Study; PETALS, Pregnancy Environment and Lifestyle Study; PrPb, propyl paraben; PrtRico, ECHO Puerto Rico cohort; TIDES, The Infant Development and Environment Study; 2,4-DCP, 2,4-dichlorophenol; 2,5-DCP, 2,5-dichlorophenol

References

1. Yoshida T, Andoh K, Fukuhara M. Urinary 2,5-dichlorophenol as biological index for p-dichlorobenzene exposure in the general population. Archives of Environmental Contamination and Toxicology. 2002;43:0481–5.

2. Dubey D, Sharma V, Pass S, Sawhney A, Stüve O. Para-dichlorobenzene toxicity - a review of potential neurotoxic manifestations. Therapeutic Advances in Neurological Disorders. 2014;7:177–87.

3. Mustieles V, Balogh RK, Axelstad M, Montazeri P, Márquez S, Vrijheid M, et al. Benzophenone-3: Comprehensive review of the toxicological and human evidence with meta-analysis of human biomonitoring studies. Environment International. 2023;173:107739.

4. Mustieles V, D'Cruz SC, Couderq S, Rodríguez-Carrillo A, Fini J-B, Hofer T, et al. Bisphenol A and its analogues: A comprehensive review to identify and prioritize effect biomarkers for human biomonitoring. Environment International. 2020;144:105811.

5. Nowak K, Ratajczak–Wrona W, Górska M, Jabłońska E. Parabens and their effects on the endocrine system. Molecular and Cellular Endocrinology. 2018;474:238–51.
